# Supplementary material for: The contribution of hospital-acquired infections to the COVID-19 epidemic in England in the first half of 2020
Source: BMC Infect Dis. 2022 Jun 18;22:556. doi: 10.1186/s12879-022-07490-4 (PMC9206097; doi:10.1186/s12879-022-07490-4)
Supplement: Supplementary file 1 — Additional file 1. Definitions. [file 12879_2022_7490_MOESM1_ESM.docx]

**Additional File 1: Definitions**

| **Term** | **Definition** | **Specifics for this analysis** |
| --- | --- | --- |
| **Case** | An individual that has COVID-19 (the disease due to SARS-CoV-2 infection) |  |
| **Patient with a hospital-acquired SARS-CoV-2 infection** | A patient with an infection acquired in the hospital setting, whether identified or not |  |
| **Identified hospital acquired infection** | An individual with a SARS-CoV-2 infection that has been identified as hospital-acquired | In this work SARS-CoV-2 infection is detected by a case with symptom onset prior to 5, 8 or 15 days from admission in line with the ECDC definition (1) |
| **unidentified hospital acquired infection** | An individual with a SARS-CoV-2 infection that has not been identified as hospital-acquired | Some of these will be misclassified as community-acquired, some will be “missed” as the patient is discharged before symptom onset. |
| **Symptom onset** | The self-reported start date of COVID-19 symptoms | Here we mostly use the CO-CIN data which has a symptom onset defined by the ISARIC protocol. |
| **Community-acquired** | A patient with an infection with SARS-CoV-2 that is classified as being acquired outside of the hospital in the community setting | Individuals with a symptom onset before the cutoff date, including before admission, are classified as community-acquired in CO-CIN. |
| **Hospital-linked** | A patient with an infection that was acquired by transmission in the community from a four-generation chain of transmission originating with a unidentified “missed” hospital-acquired infection | We assume that every hospital-acquired infection that is “missed” is discharged into the community and can cause onward transmission. We calculated the number over approximately one month after discharge (4 x 6.7 days). |
| **Classified** | The assignation of “community-acquired” or “hospital-acquired” to the infection within a hospitalised patient with COVID-19 | We use this to specify the current classification of a symptomatic infection. Hence a case could be classified as “community-acquired” but actually be “hospital-acquired”. We chose to use classified as well as “identified” as some hospital acquired infections would not have been classified whilst some would. |
| **Identified** | The detection of hospital-acquired infection |  |
| **Detection date** | the most recent of (1) date of symptom onset or (2) date of admission if this occurred after symptom onset for a patient with COVID-19, censored at date of discharge | For any “community-onset” case this was their admission date. For “hospital-onset, hospital-acquired” cases this was their date of symptom onset (Table 1). |

**Table S1: Common definitions**

**References**

1. European Centre for Disease Prevention and Control. Surveillance definitions for COVID-19 [Internet]. 2021 [cited 2021 Apr 20]. Available from: https://www.ecdc.europa.eu/en/covid-19/surveillance/surveillance-definitions
