# Supplementary material for: The contribution of hospital-acquired infections to the COVID-19 epidemic in England in the first half of 2020
Source: BMC Infect Dis. 2022 Jun 18;22:556. doi: 10.1186/s12879-022-07490-4 (PMC9206097; doi:10.1186/s12879-022-07490-4)
Supplement: Supplementary file 2 — Additional file 2. Datasets. Trust and case number differences; LoS distributions. [file 12879_2022_7490_MOESM2_ESM.docx]

**Additional File 2: Datasets**

**Trust and case number differences**

For COCIN, we included 123 Trusts and 3 super-Trusts in the final data analysis (see Additional File 4 for definition of super-Trusts, basically pooled Trusts to account for frequent transfers).

SUS covers 589 Trusts in England. 319 of these reported a total of 91,319 COVID-19 cases up to 31st July 2020. 13,415 of these cases were not included in COCIN: suggesting that COCIN has a coverage of ~85% of the total.

**CO-CIN data inclusion**

Using the 3rd December CO-CIN data extraction, there were 104,672 unique subject IDs. Of these 78% had a symptom onset and admission date. 62%, or 65,028/104,672 unique subject IDs were included in the final dataset. The included cases were those with (i) a symptom onset date, (ii) an admission date, (iii) a symptom onset date after the 12st January 2020 and (iv) a symptom onset date before the 31st July 2020. Most patients had a symptom onset before admission (Figure S1).


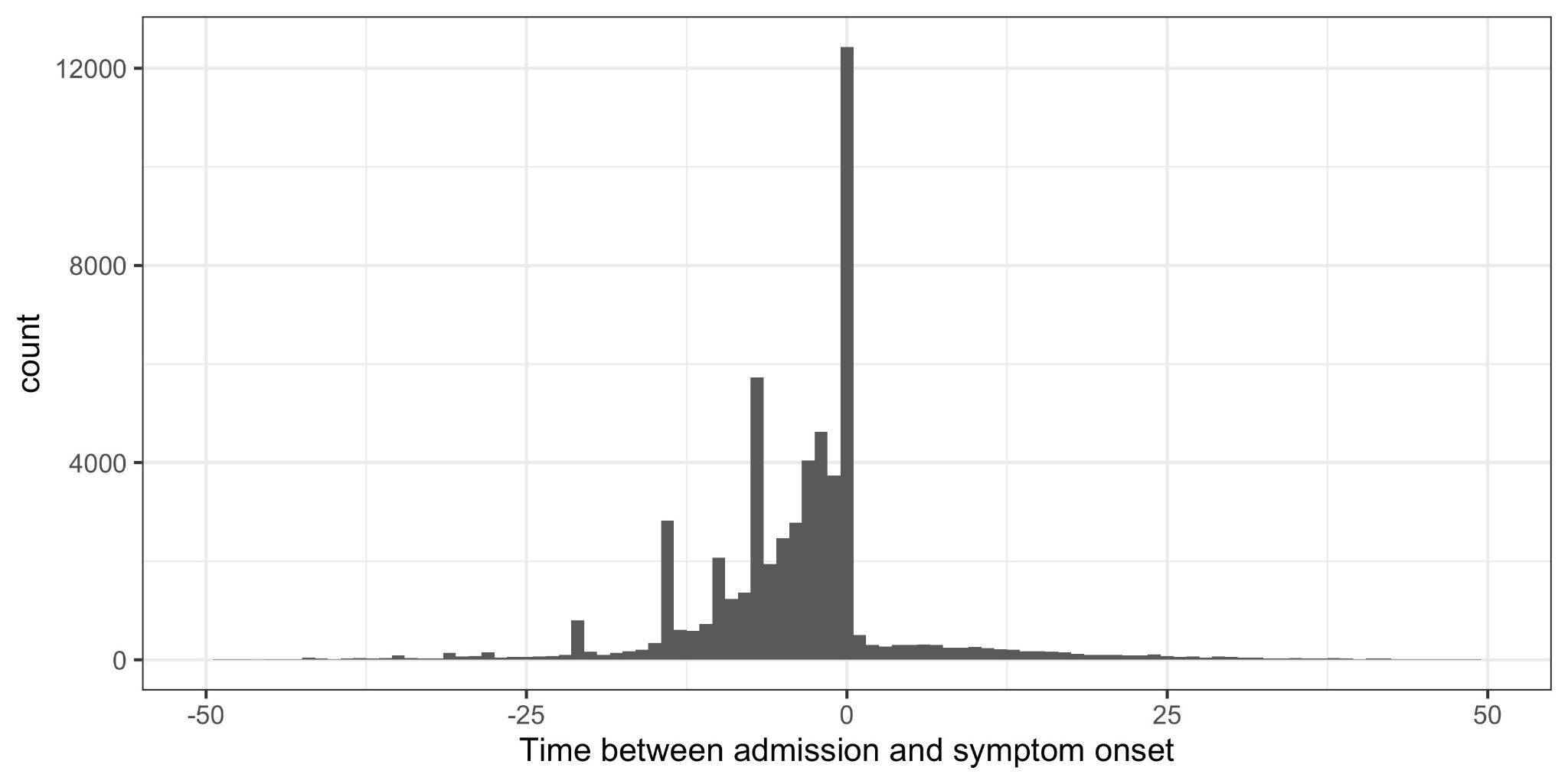


**Figure S1: Data from CO-CIN on time between admission to hospital and symptom onset.**

We defined a date of “detection” as the most recent of (1) date of symptom onset or (2) date of admission if this occurred after symptom onset for a patient with COVID-19, censored at date of discharge. For any “community-onset” case this was their admission date. For “hospital-onset, hospital-acquired” cases this was their date of symptom onset (Table 1).

**LoS distributions**

The length of stay (LoS) for non-COVID-19 positive patients is shown by week (in Figure S2) and over time (in Figure S3). Non-COVID-19 patients were defined as in-patients who never had a positive test, or who tested positive either after their hospital stay or more than 14 days before admission. Only one Trust (RX3) was removed as there were only 11 LoS data points (vs. a mean of 927 data points across other included acute Trusts) for non-COVID patients.


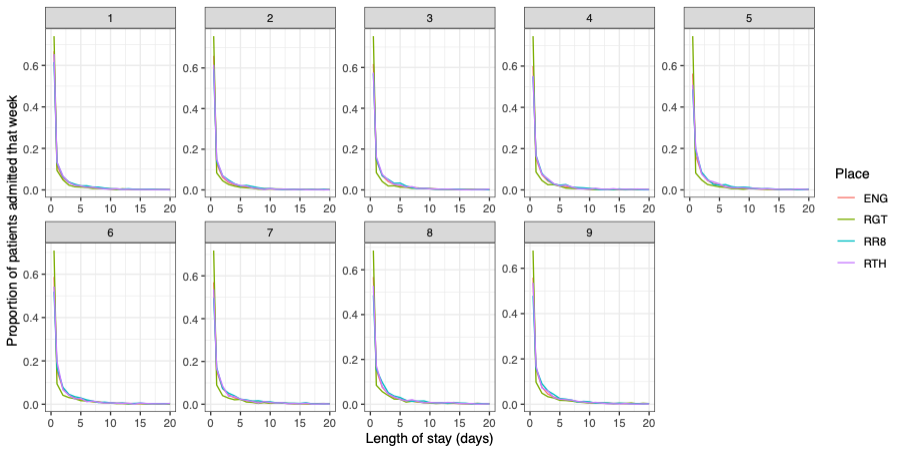


***Figure S2: Length of stay variation by week (facet) and three example Trusts (colour)***

**
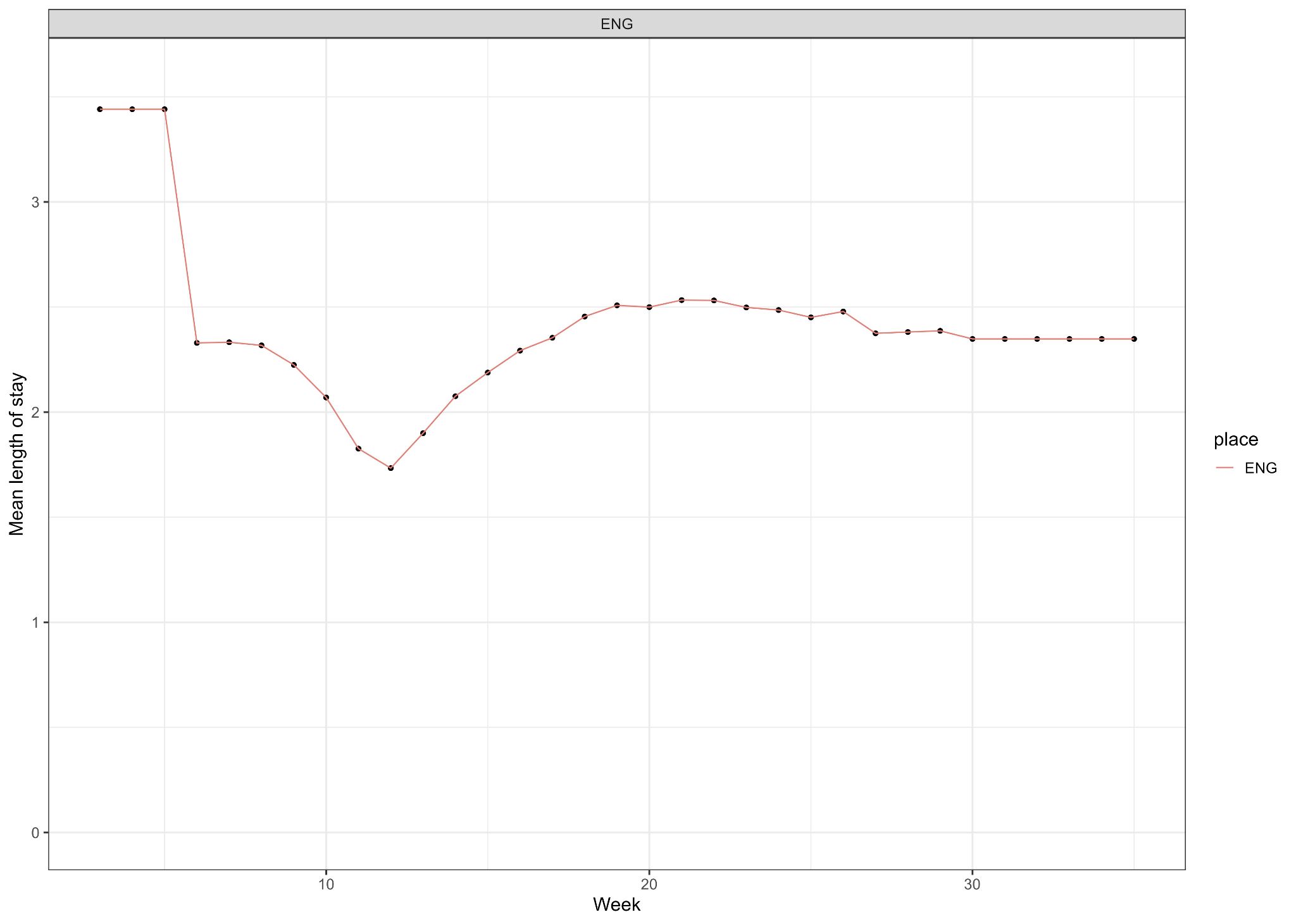

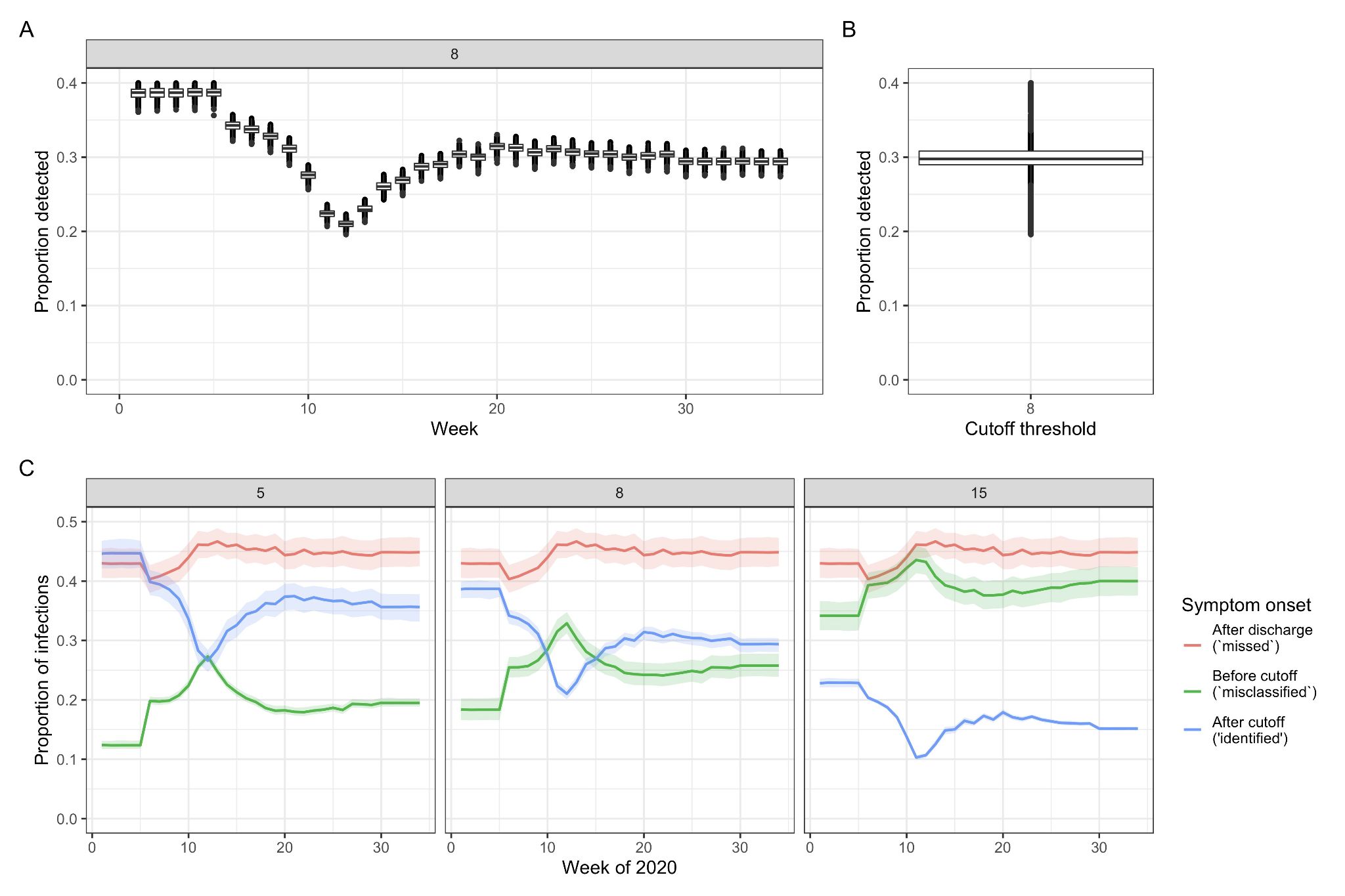
**

***Figure S3: Average length of stay over time for England in top panel to compare to proportion identified for England (equivalent to Figure 3A from main text, bottom panel)).***
