# Supplementary material for: The contribution of hospital-acquired infections to the COVID-19 epidemic in England in the first half of 2020
Source: BMC Infect Dis. 2022 Jun 18;22:556. doi: 10.1186/s12879-022-07490-4 (PMC9206097; doi:10.1186/s12879-022-07490-4)
Supplement: Supplementary file 3 — Additional file 3. Admission with infection levels. [file 12879_2022_7490_MOESM3_ESM.docx]

**Additional File 3: Admission with infection levels**

**What proportion of hospitalised patients with symptom onset after the cut-off day T had been infected in the community and admitted to hospital for a non-covid reason while latently infected?**

**Data**

The maximum prevalence of infection from seroprevalence surveys in the UK prior to September 2020 has been approximately:

0.5% from ONS (modelled, smoothed)(1)

0.3% from REACT1(2)

Between the 27^th^ April & 10^th^ May, ONS estimated prevalence of infection to be: 0.27 (0.17-0.41)%.

**Model**

The percentage of people at day T with COVID that acquired it in the community =

Prevalence of infection at entry x probability still in hospital at day T x probability symptoms developed after day T = (prev * (1-pexp(*T*,1/los)) * (1-plnorm(*T*,1.621, 0.418))*100.

Baseline measures

For example, using the ONS data for early May:

0.0027 * (1-pexp(*T*,1/los)) * (1-plnorm(*T*,1.621, 0.418))*100

For *T* > 10 this is zero due to very few patients remaining in hospital past this point (even assuming los for non-COVID of 7 days, which is an overestimate).

For *T* = 5, the value is 0.03 (0.02,0.04)%, 0.05 (0.03,0.08)% 0.07 (0.04,0.1)% for mean length of stays of 3, 5 or 7 days respectively. In conclusion < 0.1% of cases past day 5 are likely to be acquired in the community currently.

At the maximum prevalence:

At maximum prevalence 0.0054 * (1-pexp(*T*,1/los)) * (1-plnorm(*T*,1.621, 0.418))*100

For *T* > 10 this is zero due to very few patients remaining in hospital past this point (even assuming los for non-COVID of 7 days, which is an overestimate).

For *T* = 5, the value is 0.05 (0.04,0.07)%, 0.1 (0.08,0.13)% 0.14 (0.1,0.17)% for mean length of stays of 3, 5 or 7 days respectively. In conclusion < 0.2% of cases past day 5 are likely to be acquired in the community currently.

**Conclusion:** The prevalence was likely to be higher at the peak of the epidemic, but even at 10x higher this would be less than 1% of cases past day 5 being attributable to non-recent hospital transmission.

**References**

1. Office for National Statistics. Coronavirus (COVID-19) - Office for National Statistics [Internet]. 2020 [cited 2021 Feb 11]. Available from: https://www.ons.gov.uk/peoplepopulationandcommunity/healthandsocialcare/conditionsanddiseases

2. REACT Study [Internet]. 2020 [cited 2021 Feb 11]. Available from: https://www.reactstudy.org/
