# Supplementary material for: The contribution of hospital-acquired infections to the COVID-19 epidemic in England in the first half of 2020
Source: BMC Infect Dis. 2022 Jun 18;22:556. doi: 10.1186/s12879-022-07490-4 (PMC9206097; doi:10.1186/s12879-022-07490-4)
Supplement: Supplementary file 4 — Additional file 4. Comparing COCIN and SUS by week. [file 12879_2022_7490_MOESM4_ESM.docx]

**Additional file 4: Comparing COCIN and SUS by week**

There are several discrepancies between the Trusts enrolled in COCIN and SUS. The steps to calculate how to go from non-complete enrolment in CO-CIN to SUS (national COVID-19 case total data) are given below.

For each Trust in CO-CIN and each week (aggregated using lubridate::week (1)), the proportion of CO-CIN cases in SUS was calculated.

*When the proportion of SUS in CO-CIN was less than 1 (expected as CO-CIN enrolment based)*

The algorithm for a single Trust or England, for a set cutoff was

1. Calculate the weekly proportion of CO-CIN cases in SUS
2. Inverse this weekly proportion to give a multiplier
3. In the cleaned (removed those with no subject onset or admission date), one row per subject CO-CIN, enter the multiplier for the week of the admission date for each subject
4. Multiply each single hospital-acquired defined case by the multiplier for their week of admission to inflate the hospital-acquired case numbers. These were rounded to the nearest number.
5. Aggregate over individual case data to get total number of
   1. hospital-acquired cases (by summing over the inflated case numbers at the individual level)
   2. Total cases (by summing over the multipliers: each single entry needs inflating)

Code in: trust_number_noso_all.R in <https://github.com/gwenknight/hai_first_wave.git> (2).

*When the proportion of SUS in CO-CIN was greater than 1 (unexpected as SUS should have all cases)*

If this proportion was greater than 1 (i.e. unexpected more cases in CO-CIN than SUS), then we explored the actual numerical difference in case numbers that was seen. If this difference in numbers was greater than 20% of the original total numbers in CO-CIN then we explored the difference further: 20 Trusts. The idea here is that especially in May / June there is a small number of cases admitted per week (< 5). It may be that a proportion >1 is then 2 in CO-CIN but only 1 in SUS. If their relative difference is not so big (< 20%) of the original CO-CIN data then we ignore this issue and set the proportion to 1.

For those to be explored further, we looked at the impact of capping the proportion at 1 and multiplying through the CO-CIN data to match the SUS data. If the total number of cases was greater than 150% of SUS then explored these further: this was the case for 5 Trusts.

In closer investigation we found that several of these Trusts had frequent transfers with other Trusts, for example three Trusts in one county, meaning that cases may be differently labelled as being in one Trust or the other in COCIN and SUS. This may be as SUS is based on test date and COCIN on symptom onset which may occur for a patient in different Trusts. To tackle this we aggregated Trusts with frequent transfers into super-Trusts. This results in three super-Trusts (R13, RR0, ESX) which included 2 (RT3, R1K), 2 (RRF, 02H), or 3 (RDD, RQ8, RAJ) Trusts and covered four of these problem Trusts. The fifth Trust (RBA) we removed from analysis as the discrepancy was substantial: more than 20 cases in COCIN than SUS at the peak and a secondary SUS peak that was not present in CO-CIN.

The resulting proportion of CO-CIN cases in SUS over time is shown in Figure S4.

***
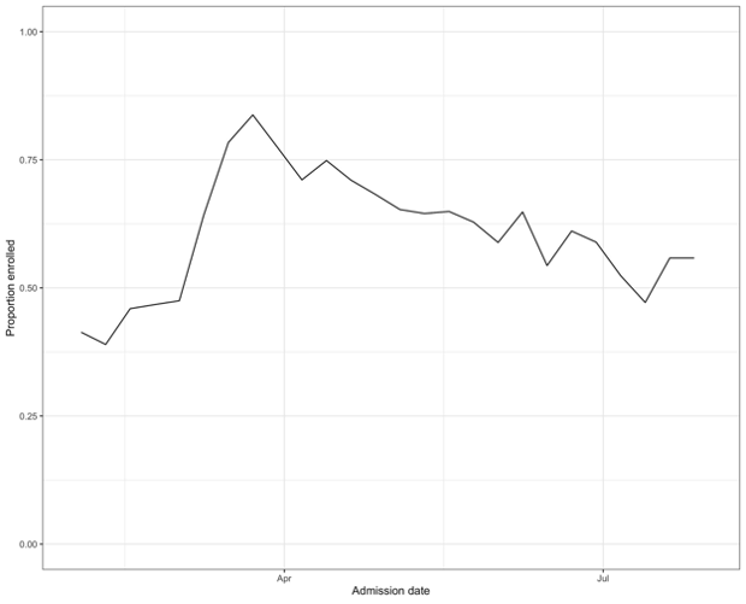
***

***Figure S4: Proportion of CO-CIN cases in SUS over time for acute English Trusts***

**References**

1. Grolemund G, Wickham H. Dates and Times Made Easy with lubridate. J Stat Softw. 2011 Apr 7;40(1):1–25.

2. Knight GM. Supporting Github repository [Internet]. Github; 2021. Available from: https://github.com/gwenknight/hai_first_wave.git
