# Supplementary material for: The contribution of hospital-acquired infections to the COVID-19 epidemic in England in the first half of 2020
Source: BMC Infect Dis. 2022 Jun 18;22:556. doi: 10.1186/s12879-022-07490-4 (PMC9206097; doi:10.1186/s12879-022-07490-4)
Supplement: Supplementary file 5 — Additional file 5. Calculations of proportion undetected hospital-acquired SARS-CoV-2 infection. [file 12879_2022_7490_MOESM5_ESM.docx]

**Additional File 5: Calculations of proportion undetected hospital-acquired SARS-CoV-2 infection**

***
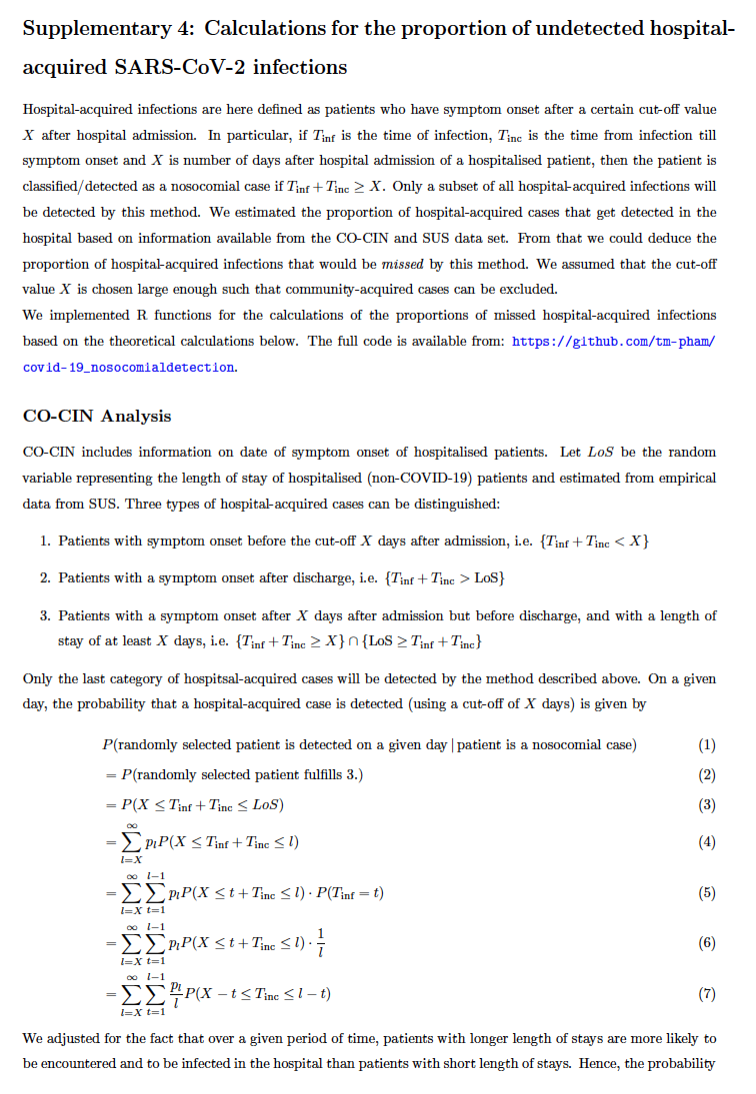
***

**
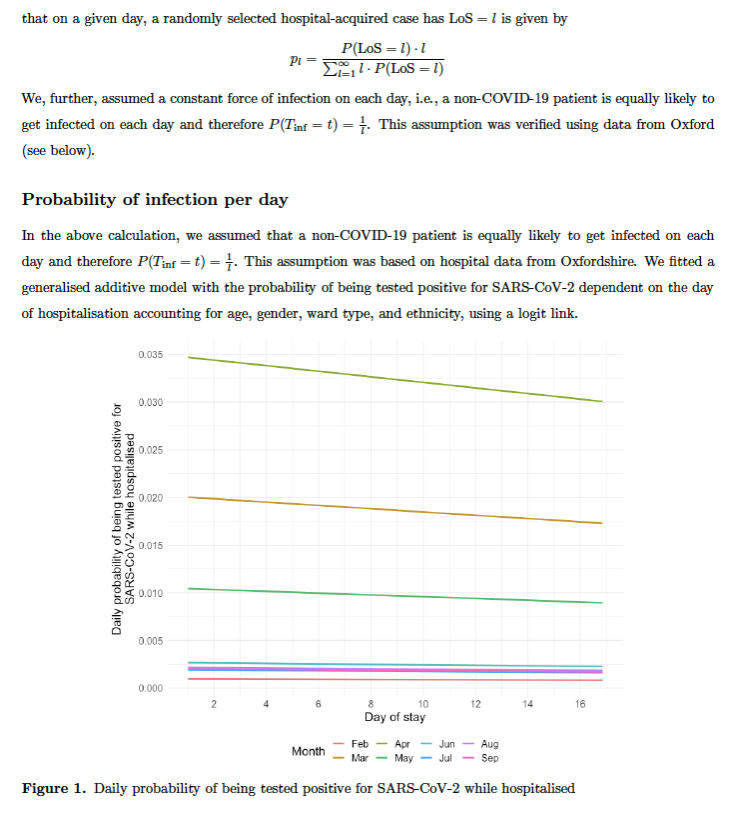
**
