# Supplementary material for: The contribution of hospital-acquired infections to the COVID-19 epidemic in England in the first half of 2020
Source: BMC Infect Dis. 2022 Jun 18;22:556. doi: 10.1186/s12879-022-07490-4 (PMC9206097; doi:10.1186/s12879-022-07490-4)
Supplement: Supplementary file 6 — Additional file 6. Parameterisation and additional methods. [file 12879_2022_7490_MOESM6_ESM.docx]

**Additional File 6: Parameterisation and additional methods**

| Parameter in R code | Definition | Literature | Notes | Base case |
| --- | --- | --- | --- | --- |
| *prop_miss_hosp* | Proportion of recently hospitalised patients with missed hospital-acquired infections that will be subsequently admitted to hospital with COVID-19 | Infection hospitalisation ratio that ranged from < 5% in those aged 40 to > 40% in those aged 80+ (1) | Multiplying the proportion of the non-COVID hospital population in each age group by the risks in Knock et. al. leads to an upper estimate of 15%. These patients have previously been hospitalised so have a higher risk of re-infection than others in their same age group.  We assumed a uniform distribution between 10% and 15%. For each patient a Bernouilli trial then used this sample to assess whether the patient would return | unif(0.1,0.15) |
|  |  | 3,4,8,12,17,18% infections are hospitalised for 10yr age groups from 30 to 80+ respectively (Table 3, (2)) |  |  |
|  |  | Non-COVID hospital population composed of 33% older than 70, 60% older than 50 (5 yr age group data used) (3) |  |  |
| *prop_comm_hosp* | Proportion of community infections that will be hosp. cases of COVID-19 | 3.5% (95% CrI 3.3%-3.7%) of people infected needed hospitalisation (1). This is approximated here by the normal distribution which gives a 95% CrI of 3.4%-3.6%. | Assume normal distribution with mean from literature, and estimated standard deviation to match range | norm(0.035, 0.0005) |
| *time_inf_to_symp_mean* | Time to symptom onset from infection | Incubation period: mean of 5.1 days (4) | Use the Lauer distribution for analysis | 1.62 |
| *time_inf_to_symp_sd* |  | Log normally distribution, with mean of 5.8 (95% CI 5.0 to 6.7) days (5) |  | 0.4 |
| *time_inf_to_symp_mean_sd* | Standard deviation in estimates of mean and standard deviation time to symptom onset from infection | Incubation period: mean of 5.1 days (4) | Taken from range from Lauer et al | 0.064 |
| *time_inf_to_symp_sd_sd* |  |  |  | 0.0691 |
| *time_symp_to_hosp_meanlog* | Time to hospitalisation from symptom onset | Gamma distribution, shape parameter equal to the mean of 7 days (standard deviation 2.65) “ (6) | Scenario (1) : Log normal distribution fitted to CO-CIN data (mean of 7 days, median 6 days)  Scenario (2): Gamma distribution as in Davies et al (gamma(7,1))  Scenario (3): Log-normal distribution from FF100 data (log-normal(1.44, 0.72))  (See below) | 1.66 |
| *time_symp_to_hosp_sdlog* |  |  |  | 0.89 |
|  |  | Analysis of “first” wave CO-CIN gives a mean of 7.7 days, median = 6 days and a range of 1-129. |  |  |
|  |  | Analysis of first few 100 cases in the UK (7) |  |  |
| Time from symptom onset to hospitalisation | | Approximately 2 weeks | Sum of means = 7 + 5.1 = 12.1 days | |
| *R* | Average number of secondary infections from one infected individual in the community | Use the time varying estimates of *R* from *epiforecasts.io* as well as constants | Constant or time varying estimate | 0.8, 1.2 and “rt” |
| *infectious_shape* | Time period over which an infected individual is infectious (time from exposure to infection) | Duration of clinical infectiousness: gamma (shape=3.5, scale = 4) (6) | Taken to be as in Davies et al as a balance between underestimating due to lack of pre-clinical period vs. an overestimate if look at serial interval estimates (3 - 6 days (8)) | 4 |
| *infectious_scale* |  | Generation time estimates: 3.95 to 5.20 days (8) |  | 0.875 (3.5/4) |
| *cut-off_date* | Days from admission cut off for defining hospital-acquired case | Assumed 5, 8, 10, 14 | Will affect time series of hospital-acquired cases | 5 |

*Table S2:* Parameters values used in the model. Extended version of Table 2.

**Serial interval:**

Latency period mean of 5.1 days

Infectious period mean of 3.4 days

Subsequent infection mean of 5.1 + uniform(0,1) * 3.4 = mean of 6.8 days

For each infection, a latency period, infectious period and uniform random number were sampled. An “*R*” number of subsequent infections were then generated at a time latency period plus the uniform random number times the infectious period.

We chose to look at approximately the first month of transmission after discharge to limit the number of onward cases. It is likely that chains of transmission are short: 4 generations in China (9), and suggested to be short from genomic data in the UK and New Zealand (10,11).

**Additional methods**

Extending the methods given in the main paper we include further details for some of the stages in Figure 2 below.

1. **Proportion of hospital-acquired infections that are identified**

To calculate this we assumed that the daily risk of infection did not change with the day of hospital stay, supported by data analysis (Additional File 5). The proportions of true hospital-acquired infections which are identified is dependent on (i) the assumed cut-off threshold and (ii) the length of stay (LoS) distribution for patients hospitalised for reasons other than COVID-19 and hence at risk of becoming infected, with the latter varying by week and setting.

1. **Reclassifying community-acquired as hospital-acquired**

To determine the contribution of unidentified hospital-acquired infections to hospitalised patient burden, we estimated when an unidentified “missed'' hospital-acquired infection would return as a hospital admission by generating the entire disease progression trajectory for each unidentified “missed” hospital-acquired infection (Figure 2).

For the disease progression trajectory, the proportion returning to hospital was sampled using a Bernouilli trial and varied for each simulation (Table 2). For each individual that was expected to become a hospitalised case we sampled a time (i) from infection until discharge (ii) from infection to symptoms and (iii) from symptoms to potential hospitalisation (Figure 2, Table 2). The time since infection was subtracted from the time to hospitalisation (the sum of time to symptoms from infection and time from symptoms to hospitalisation) to calculate the time at which the unidentified “missed” hospital-acquired infected individuals would be identified but currently misclassified as a “community” case at hospital admission (new “community onset, hospital-acquired” cases, Figure 2, Table 1).

1. **Hospital-linked cases**

To account for onward transmission in the community from patients with unidentified “missed” hospital-acquired infections (due to symptom onset after discharge) we estimated "hospital-linked infections'': calculated as first-, second-, third- and fourth-generation infections. This is approximately the number of infections caused within one month after discharge (~6.7 day serial interval, Additional File 6) and assumes that most onward transmission chains are relatively short (9–11).

The time series for these was calculated by sampling a certain time to infection (a sum of a sample from the latency distribution and a sample from a uniform distribution on 0-1 multiplied by a sample from the distribution for the duration of clinical infectiousness (~ 3 days)), a number of secondary infections (using estimates for the reproduction number, *R*), a sampled proportion which progress to disease, a sampled proportion of infections that become hospitalised and a sampled time to hospitalisation (with different distributions for each symptom onset to hospitalisation scenario) (Figure 2, Table 2).

For the onward transmission, we explored three reproduction number values: a constant value of 0.8 or 1.2 with a range generated as +/- 5% of the constant value. For a time-varying estimate “*Rt”* we took upper/lower bounds for the 50% credible interval from a publicly available repository (12) (Additional File 9). Mean and 95% ranges for onward transmission infections and case numbers are presented as over the 600 simulations generated from 200 simulations on each *R* value (estimate, upper and lower bound).

1. **Reclassifying community-acquired to hospital-acquired**

The number of unadjusted identified hospital-acquired COVID-19 cases is from the inflated CO-CIN dataset (“hospital-onset, hospital-acquired” cases, Figure 2, Table 1). The unadjusted community-acquired classifications were then defined as the difference between the total number of COVID-19 hospital admissions and the unadjusted identified hospital-acquired COVID-19 cases.

We adjusted the number of hospital-acquired cases by adding our model estimates of (1) "community-onset, hospital-acquired" and (2) any hospital-linked cases, to the identified hospital-acquired case numbers ("adjusted" hospital-acquired assignations). The "adjusted" community-acquired classifications are then altered accordingly. We then calculated the proportion of community cases that were reassigned as (unadjusted community # - adjusted community #) / (unadjusted community #).

To calculate the counterfactual of no transmission in hospital settings, we compared the original total number of hospitalised cases to the adjusted community number (i.e. those that we did not model as being acquired-in or linked-to hospital settings).

**Total English burden**

Acute Trusts in CO-CIN covered approximately 85% of the COVID-19 cases recorded in SUS. In order to give estimates for all English trusts, we multiplied our results by 1.17 and assumed similar levels of nosocomial transmission in non-acute English trusts.

**References**

1. Knock ES, Whittles LK, Lees J. Report 41 - The 2020 SARS-CoV-2 epidemic in England: key epidemiological drivers and impact of interventions [Internet]. Imperial College London. 2020 [cited 2021 Mar 8]. Available from: https://doi.org/10.25561/85146

2. Verity R, Okell LC, Dorigatti I, Winskill P, Whittaker C, Imai N, et al. Estimates of the severity of coronavirus disease 2019: a model-based analysis. Lancet Infect Dis. 2020 Jun 1;20(6):669–77.

3. Hospital Admitted Patient Care Activity, 2015-16 [Internet]. NHS Digital. 2018 [cited 2021 Mar 8]. Available from: https://digital.nhs.uk/data-and-information/publications/statistical/hospital-admitted-patient-care-activity/2015-16

4. Lauer SA, Grantz KH, Bi Q, Jones FK, Zheng Q, Meredith HR, et al. The Incubation Period of Coronavirus Disease 2019 (COVID-19) From Publicly Reported Confirmed Cases: Estimation and Application. Ann Intern Med. 2020 May 5;172(9):577–82.

5. McAloon C, Collins Á, Hunt K, Barber A, Byrne AW, Butler F, et al. Incubation period of COVID-19: a rapid systematic review and meta-analysis of observational research. BMJ Open. 2020 Aug 16;10(8):e039652.

6. Davies NG, Kucharski AJ, Eggo RM, Gimma A, Edmunds WJ, Jombart T, et al. Effects of non-pharmaceutical interventions on COVID-19 cases, deaths, and demand for hospital services in the UK: a modelling study. Lancet Public Health. 2020 Jul 1;5(7):e375–85.

7. Boddington NL, Charlett A, Elgohari S, Walker JL, McDonald HI, Byers C, et al. COVID-19 in Great Britain: epidemiological and clinical characteristics of the first few hundred (FF100) cases: a descriptive case series and case control analysis. medRxiv. 2020 May 22;2020.05.18.20086157.

8. Griffin J, Casey M, Collins Á, Hunt K, McEvoy D, Byrne A, et al. Rapid review of available evidence on the serial interval and generation time of COVID-19. BMJ Open. 2020 Nov 1;10(11):e040263.

9. Luo C, Ma Y, Jiang P, Zhang T, Yin F. The construction and visualization of the transmission networks for COVID-19: A potential solution for contact tracing and assessments of epidemics. Sci Rep. 2021 Apr 21;11(1):8605.

10. Geoghegan JL, Ren X, Storey M, Hadfield J, Jelley L, Jefferies S, et al. Genomic epidemiology reveals transmission patterns and dynamics of SARS-CoV-2 in Aotearoa New Zealand. Nat Commun. 2020 Dec 11;11(1):6351.

11. Plessis L du, McCrone JT, Zarebski AE, Hill V, Ruis C, Gutierrez B, et al. Establishment and lineage dynamics of the SARS-CoV-2 epidemic in the UK. Science. 2021 Feb 12;371(6530):708–12.

12. Abbott S, Hellewell J, Thompson RN, Sherratt K, Gibbs HP, Bosse NI, et al. Estimating the time-varying reproduction number of SARS-CoV-2 using national and subnational case counts. Wellcome Open Res. 2020 Dec 8;5:112.
