# Supplementary material for: The contribution of hospital-acquired infections to the COVID-19 epidemic in England in the first half of 2020
Source: BMC Infect Dis. 2022 Jun 18;22:556. doi: 10.1186/s12879-022-07490-4 (PMC9206097; doi:10.1186/s12879-022-07490-4)
Supplement: Supplementary file 7 — Additional file 7. Symptom onset to hospitalisation. [file 12879_2022_7490_MOESM7_ESM.docx]

**Additional File 7: Symptom onset to hospitalisation**

As this was a key parameter for our estimates we chose to perform a scenario analysis around this distribution.

*Baseline scenario 1: “Best” fit to CO-CIN raw and smoothed data*

With data on 38,168 patients from CO-CIN reporting a symptom onset prior to hospitalisation in Wave 1, we could estimate the best fit to the data. However, the data suffered from “heaping” issues where patients preferably reported symptom onset data 1 week, 10 days, a fortnight or 3 weeks before hospital admission (Figure S6). This has been seen for many types of participant reported data (e.g. income (1)). To account for this we fitted to (1) the raw data (Figure S6) below using the *fitdistr R* package (2) and (2) used a penalized composite link model (3,4) to adjust for this heaping. We then compared the model fits using the Akaike Information Criterion (AIC) (5).

For both fitting to the raw and smoothed data the distribution with the smallest AIC value was the log-normal distribution (orange line in both Figure S6 and S7): AIC for the gamma distribution (next smallest AIC) was 228080 and 229646 for the smoother or raw data respectively, whilst for the log-normal distribution it was 225675 and 226842.

The values for the log-normal distribution fitted to the raw were:

Meanlog 1.662 (0.005) SDlog 0.889 (0.003)

And smoothed data:

Meanlog 1.665 (0.005) SDlog 0.894 (0.003)

We used a lognormal(1.66, 0.89) distribution in the base case Scenario 1.

*Scenario 2: previous estimates*

We also took a scenario which used a previous estimate of the time from symptom onset to hospitalisation as a gamma distribution with shape 7 and rate 1 (6) (grey line in Figure S6). This was calculated using international data from the first wave (7,8).

*Scenario 3: First Few 100 (FF100) cases in Great Britain*

We used data from the first few 100 cases data from Public Health England (9). This contains information on symptoms from the first 492 cases, 167 of which were hospitalised. At this time there was not a strict list of symptoms as there was later in 2020 (loss of taste / smell, continuous cough, fever). Fitting to this data suggested a best fit of logNormal distribution with mean log = 1.44, SD log = 0.72.

**
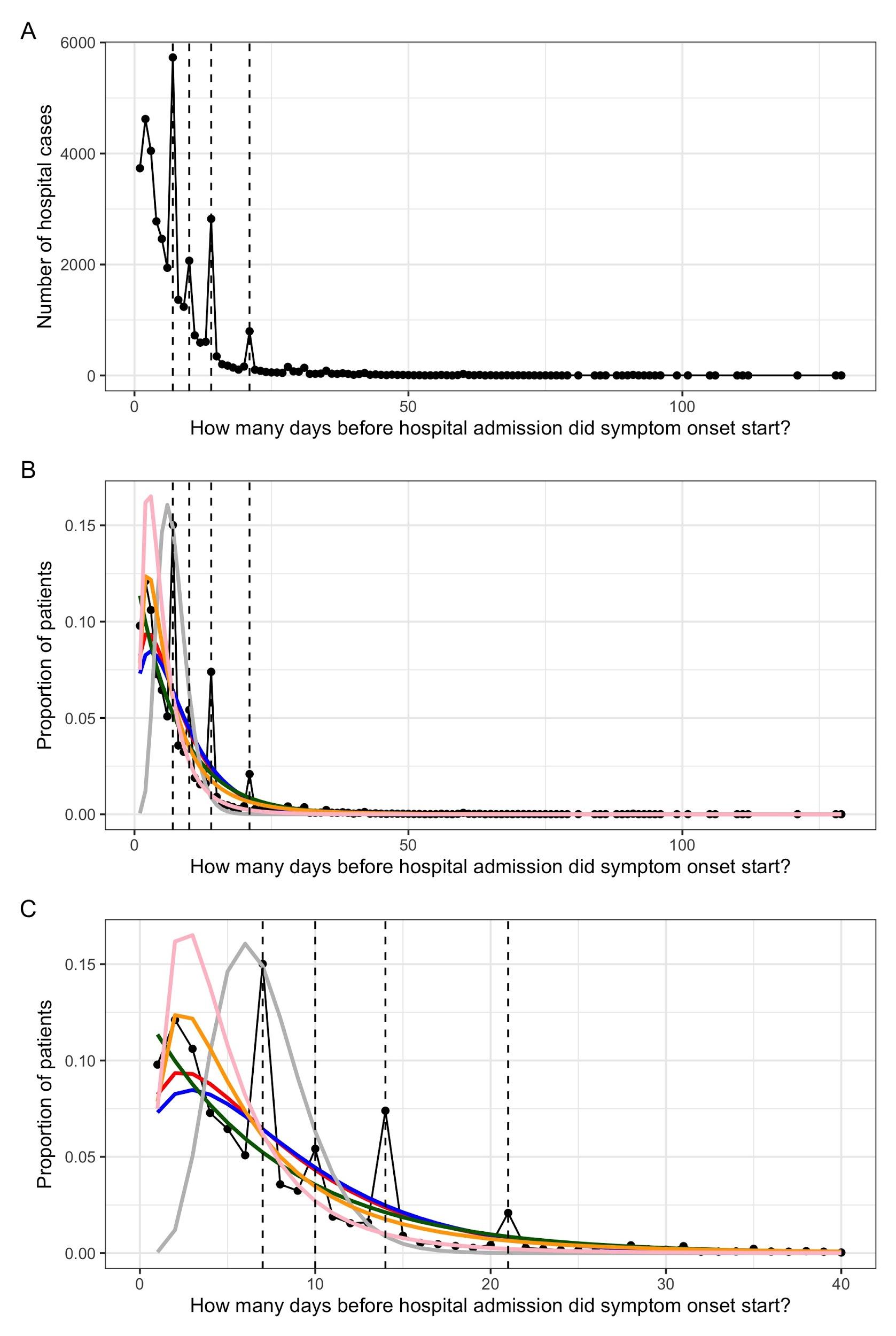
**

**Figure S6: What is the distribution of symptom onset before hospitalisation? (A) CO-CIN data for 38,168 patients from Wave 1 in England with a symptom onset and hospital admission date. Dashed lines indicate heaps in the data at 7, 14, 10 and 21 days prior to admission. (B) Results of probability distribution fitting to the data: red = gamma, blue = negative binomial, dark green = exponential, orange = log-normal (Scenario 1). The grey line is the distribution from** (6) **(Scenario 2: ~gamma(7,1)) and the pink line is the distribution from the FF100 data (Scenario 3: lognormal (1.44, 0.72) (C) Zoom in on (B) to show smaller differences in days between symptom onset and admission.**

**
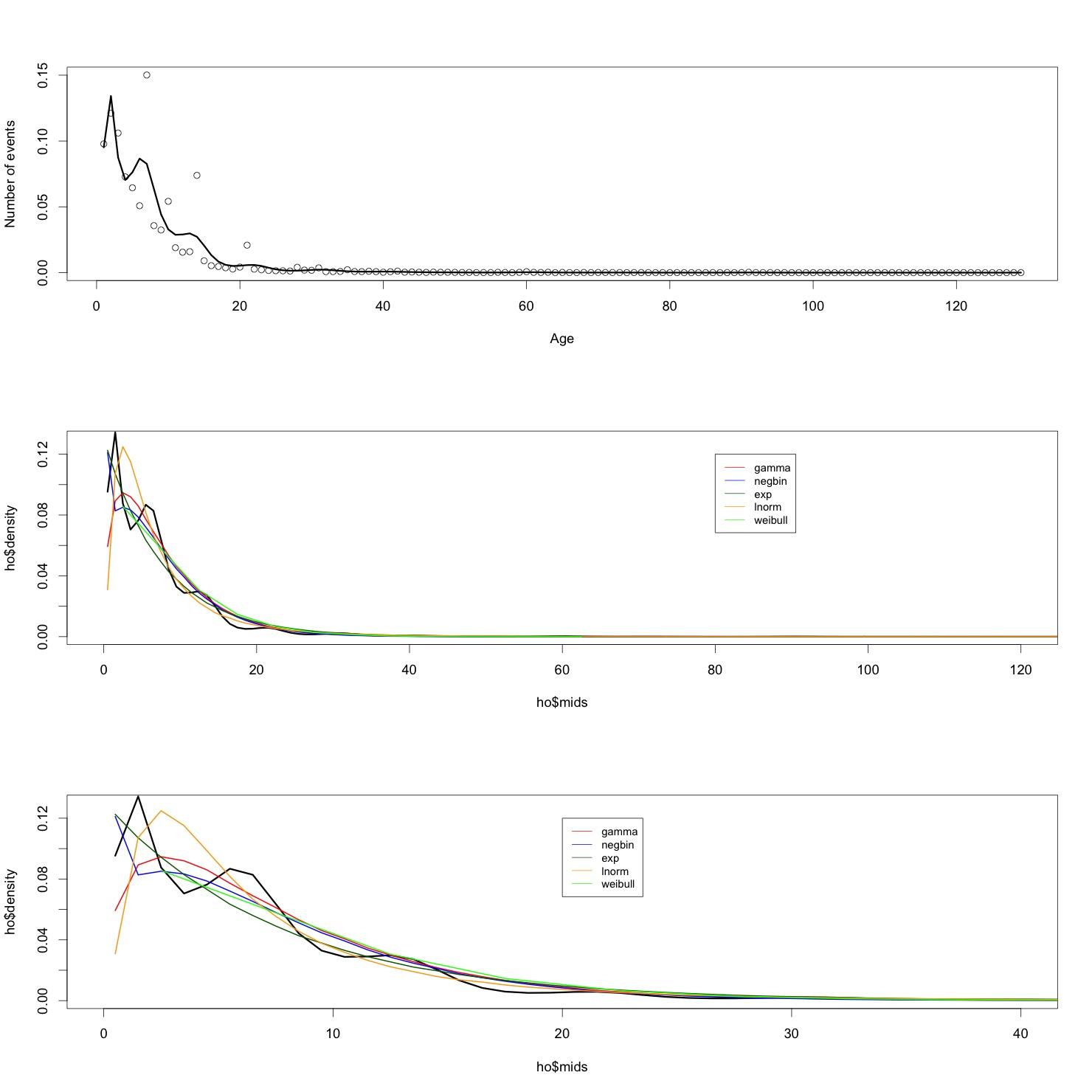
**

**Figure S7: What is the distribution of symptom onset before hospitalisation? (A) CO-CIN data (dots) smoothed using a penalized composite link model to give the black line. (B) Results of probability distribution fitting to the smoothed data (black line) (C) Zoom in on smaller differences between symptom onset and admission.**

**References**

1. Zinn S, Würbach A. A statistical approach to address the problem of heaping in self-reported income data. J Appl Stat. 2016 Mar 11;43(4):682–703.

2. Delignette-Muller ML, Dutang C. fitdistrplus: An R Package for Fitting Distributions. J Stat Softw. 2015 Mar 20;64(1):1–34.

3. Rizzi S, Gampe J, Eilers PHC. Efficient estimation of smooth distributions from coarsely grouped data. Am J Epidemiol. 2015 Jul 15;182(2):138–47.

4. Rizzi S, Thinggaard M, Engholm G, Christensen N, Johannesen TB, Vaupel JW, et al. Comparison of non-parametric methods for ungrouping coarsely aggregated data. BMC Med Res Methodol. 2016 May 23;16(1):59.

5. Akaike H. A new look at the statistical model identification. IEEE Trans Autom Control. 1974 Dec;19(6):716–23.

6. Davies NG, Kucharski AJ, Eggo RM, Gimma A, Edmunds WJ, Jombart T, et al. Effects of non-pharmaceutical interventions on COVID-19 cases, deaths, and demand for hospital services in the UK: a modelling study. Lancet Public Health. 2020 Jul 1;5(7):e375–85.

7. Linton NM, Kobayashi T, Yang Y, Hayashi K, Akhmetzhanov AR, Jung S mok, et al. Incubation Period and Other Epidemiological Characteristics of 2019 Novel Coronavirus Infections with Right Truncation: A Statistical Analysis of Publicly Available Case Data. J Clin Med [Internet]. 2020 Feb 17 [cited 2021 Mar 19];9(2). Available from: https://www.ncbi.nlm.nih.gov/pmc/articles/PMC7074197/

8. Cao B, Wang Y, Wen D, Liu W, Wang J, Fan G, et al. A Trial of Lopinavir–Ritonavir in Adults Hospitalized with Severe Covid-19. N Engl J Med. 2020 May 7;382(19):1787–99.

9. Boddington NL, Charlett A, Elgohari S, Walker JL, McDonald HI, Byers C, et al. COVID-19 in Great Britain: epidemiological and clinical characteristics of the first few hundred (FF100) cases: a descriptive case series and case control analysis. medRxiv. 2020 May 22;2020.05.18.20086157.
