# Supplementary figures and images for: The contribution of hospital-acquired infections to the COVID-19 epidemic in England in the first half of 2020
Source: BMC Infect Dis. 2022 Jun 18;22:556. doi: 10.1186/s12879-022-07490-4 (PMC9206097; doi:10.1186/s12879-022-07490-4)

**Additional File 8: Infection to discharge calculations**

**
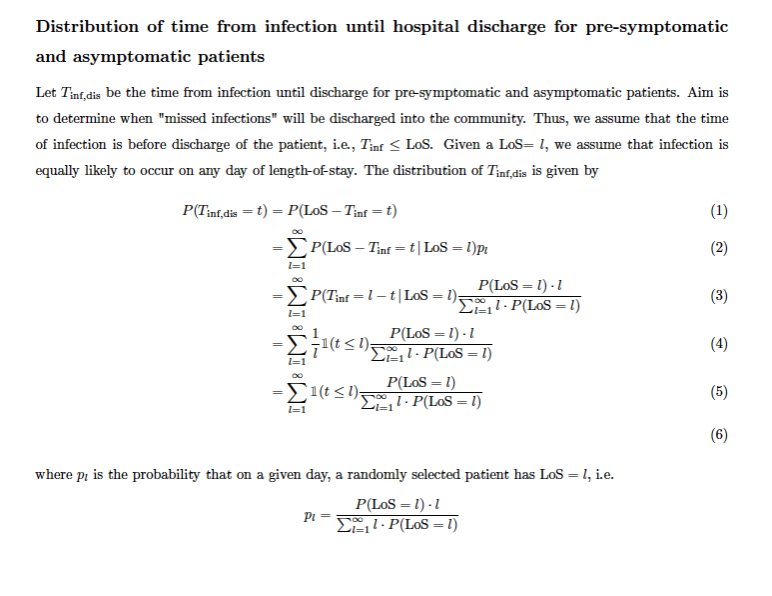
**

Supplement: Supplementary file 8 — Additional file 8. Infection to discharge calculations. [file 12879_2022_7490_MOESM8_ESM.docx]
