# Supplementary material for: The contribution of hospital-acquired infections to the COVID-19 epidemic in England in the first half of 2020
Source: BMC Infect Dis. 2022 Jun 18;22:556. doi: 10.1186/s12879-022-07490-4 (PMC9206097; doi:10.1186/s12879-022-07490-4)
Supplement: Supplementary file 9 — Additional file 9. Rt estimates. [file 12879_2022_7490_MOESM9_ESM.docx]

**Additional File 9: Rt estimates**

***
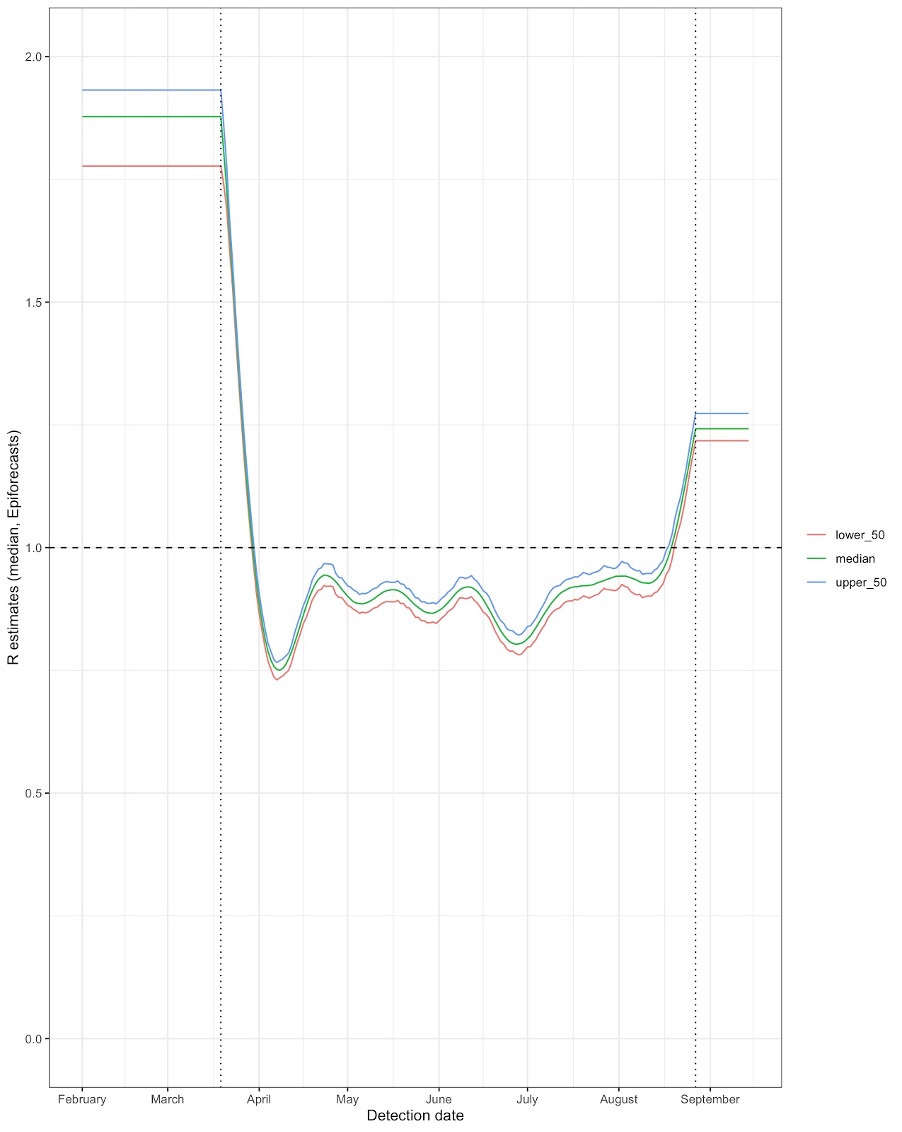
***

***Figure S8: Time varying estimate of Rt taken from EpiForecast team : median estimated using hospitalised cases* (1) *with upper and lower bounds of the 50% credible intervals.***

Uncertainty in the simulations was generated by taking the mean and 95% ranges for onward transmission infections and case numbers are presented as over the 600 simulations generated from 200 simulations on each *R* value (estimate, upper and lower bound).

**References**

1. Abbott S, Hellewell J, Thompson RN, Sherratt K, Gibbs HP, Bosse NI, et al. Estimating the time-varying reproduction number of SARS-CoV-2 using national and subnational case counts. Wellcome Open Res. 2020 Dec 8;5:112.
