# Supplementary material for: The contribution of hospital-acquired infections to the COVID-19 epidemic in England in the first half of 2020
Source: BMC Infect Dis. 2022 Jun 18;22:556. doi: 10.1186/s12879-022-07490-4 (PMC9206097; doi:10.1186/s12879-022-07490-4)
Supplement: Supplementary file 10 — Additional file 10. Uncertainty inclusion. [file 12879_2022_7490_MOESM10_ESM.docx]

**Additional File 10: Uncertainty inclusion**

200 simulations were generated. Each simulation included uncertainty from three stages:

Stage 1

As we generated estimates of the proportion identified by place and week, we included uncertainty from two elements each week:

- 1. Length of stay distribution: bootstrap the distribution for that week from SUS. As there are so many patients (n = 237,981) in the data there is little variation produced by this variation (see Figure S9 below, top left).
  2. Incubation period: sampled the parameters for the incubation period distribution (i.e. sample from the mean and standard deviation for the lognormal distribution from a normal distribution with the estimated mean and sd to give a different distribution for each sample for the time to symptom onset from infection, see Table 2).

This incubation period distribution and length of stay for non-COVID patients was used for the entire of the simulation. This is coded in “trust_proportion_detect_by_week_all.R” (1). It gives the variation in the proportion of hospital-acquired infections identified and is presented in Figure 3c, and shown again in Figure S9 for a cutoff of symptom onset more than 7 days from admission.


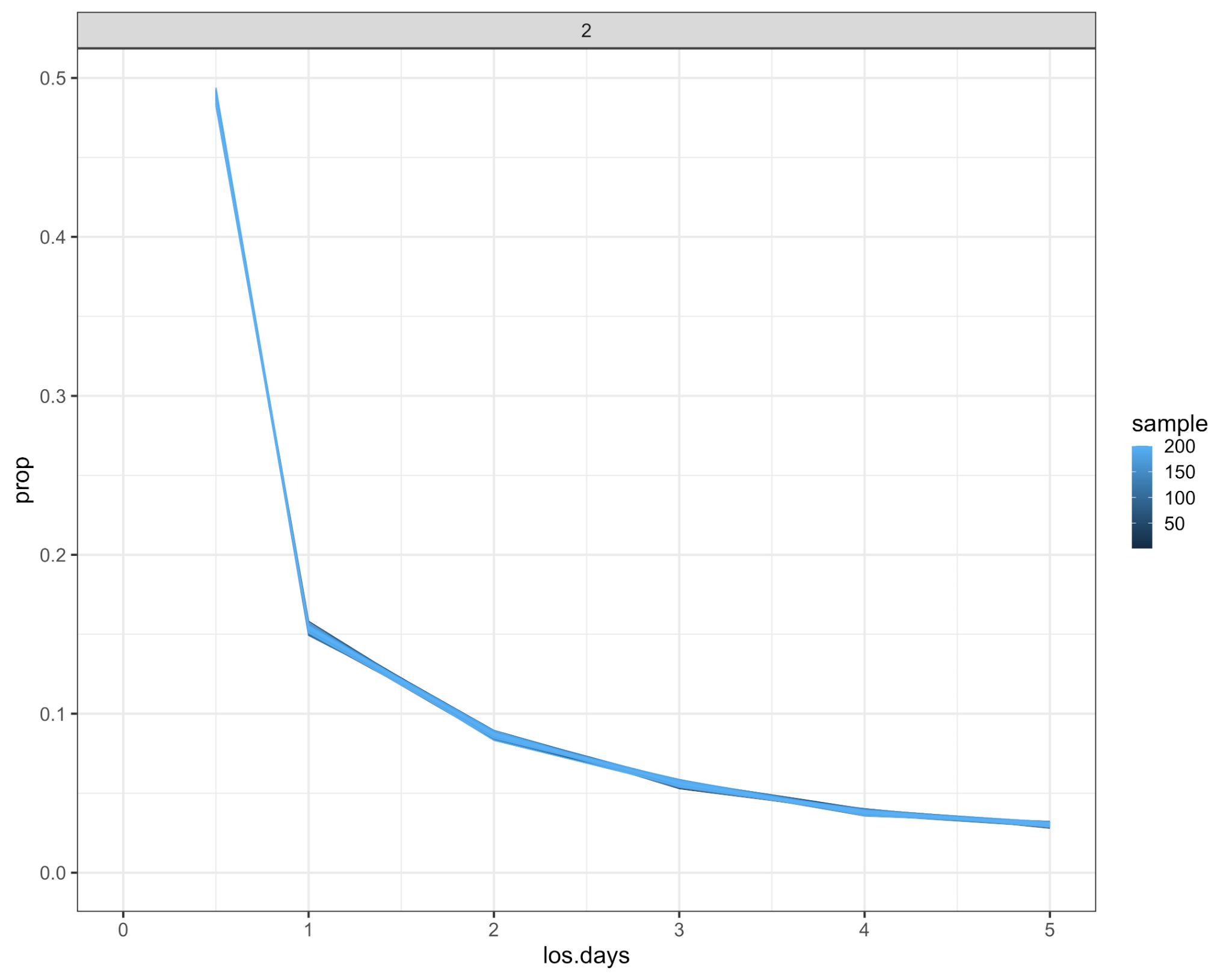

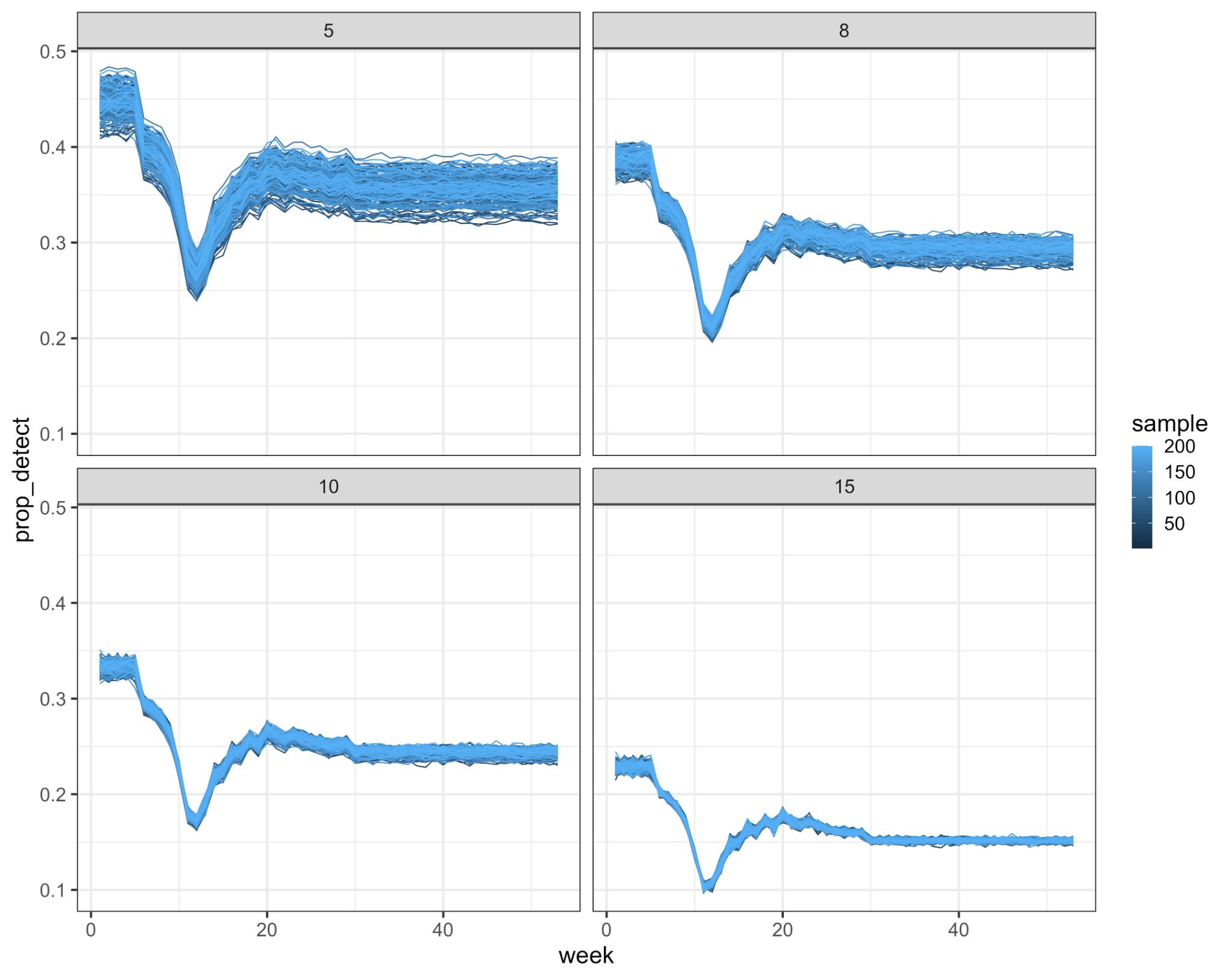


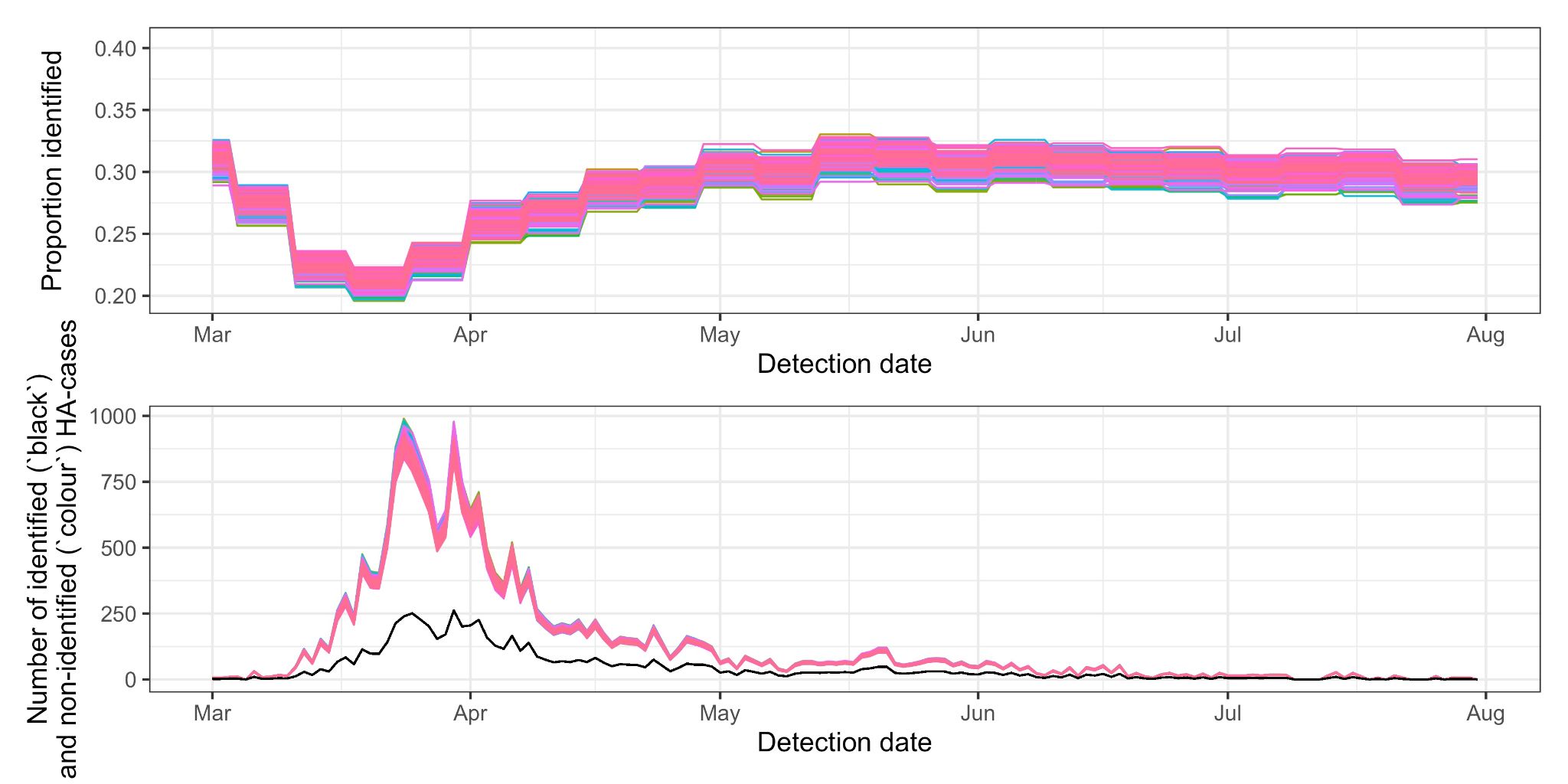


**Figure S9:** Uncertainty in the length of stay (top left) and incubation period drive uncertainty in the proportion identified (top right and middle). The inverse of this proportion multiplies the number of identified hospital-acquired cases per week (black, bottom) to calculate the number of unidentified infections (colour, bottom).

For example, towards the end of March: 250 hospital-acquired cases were identified in the inflated CO-CIN (Figure S9, bottom). At this stage it is likely that we were identifying between 20% and 22% of hospital-acquired cases (Figure S9, top). Hence this corresponds to between 840 and 1,000 missed cases.

Stage 2

To accounting for binomial sampling variation, the proportion identified for each sample and week (generated above) were used within a Bayesian framework as the binomial probability of identification to infer from the number of identified hospital-acquired cases, the total number of hospital-acquired infections (“trials”).

In more detail - using the distributions in step 1 within our function we could generate 200 samples of the proportion of true hospital acquired infections that were identified each week, *i*, and setting, *j*, from hospital data (*p_i,j_*). Assuming the number of hospital acquired infections were binomially distributed, we estimated the weekly number of true hospital-acquired infections, X*_i,j_* ~ Bin(N*_i,j_*, p*_i,j_*). Subtracting from this the identified weekly hospital-acquired infection numbers we can estimate the number of unidentified hospital-acquired infections.

This used the function in “binom_posterior.R” (1)**.**

Stage 3

The uncertainty in the natural history trajectory for each of these unidentified hospital-acquired infections was then calculated (as shown in Figure 2d) by sampling from the relevant distributions for the probability (e.g. of returning as a hospitalised cases) and timings (e.g. symptom onset after infection). This is coded in “perc_contribution_function_trust_week.R” (1).

For each unidentified infection, the probability of returning as a COVID-19 case to hospital is a Bernoulli trial for each missed infection with weekly randomly sampled probability of returning taken from a uniform distribution over 10-15%. This probability of a “missed” unidentified infection returning of a community infection becoming hospitalised is fixed across each of the 200 simulations.

Each of the following timings for each returning to hospital as a case unidentified hospital-acquired infection are then sampled from the relevant distributions (Table 2):

1. From infection to discharge
2. From infection to symptom onset (incubation period)
3. From symptom onset to hospitalisation (this is scenario dependent)

**
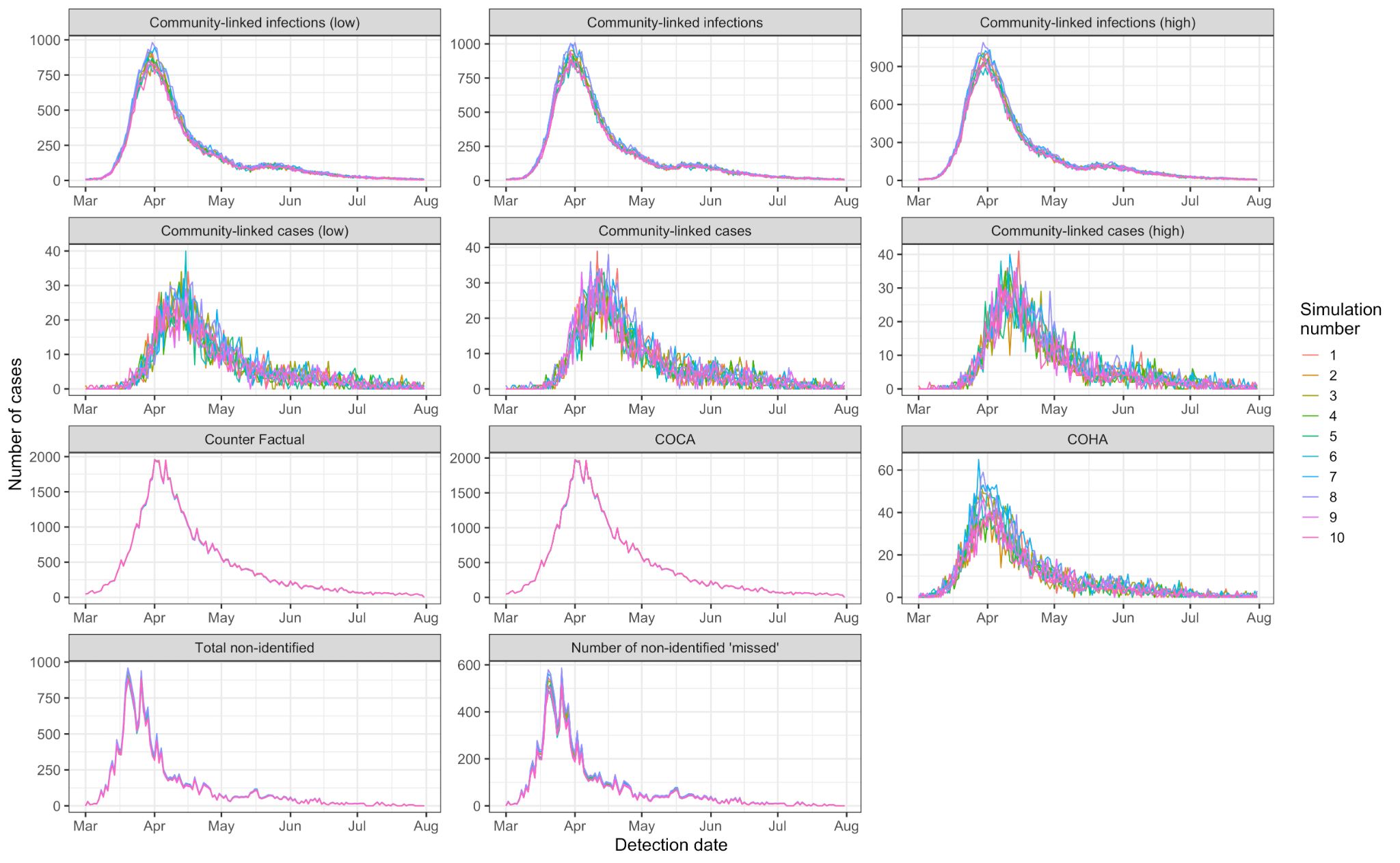
**

**Figure S10:** Example time series with a cutoff of at least 7 days from symptom onset to hospitalisation for defining a hospital-acquired case: the first 10 simulations for key model outputs are shown in the 10 colours in the above facets over time (detection date). The top two rows show the variation in community-onset, hospital-linked infections (first row) and subsequent cases (second row) at low, mean and high values of onward transmission (*R =* 0.76, 0.8, 0.84). The third row shows the counterfactual: the number of hospitalised cases there would be predicted to be without any hospital-acquisition of SARS-CoV-2, alongside the community-onset, community-acquired (“COCA”) and community-onset, hospital-acquired (“COHA) case estimates. The final row shows the same variation shown in Figure S8: the total number of unidentified infections and the “missed” subset of these (“missed” due to discharge prior to symptom onset).

**
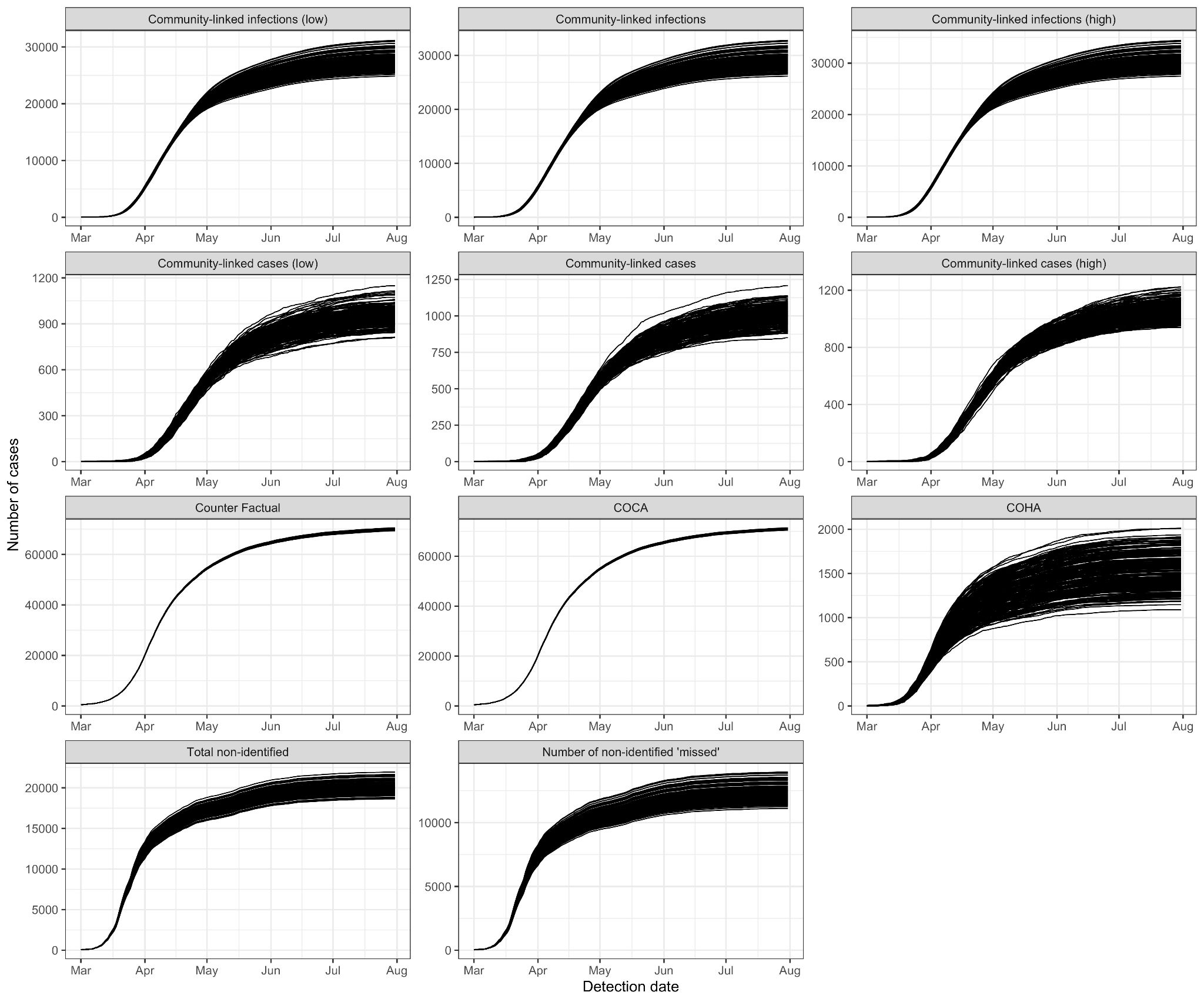
**

**Figure S11:** Example cumulative values as in Figure S10 for all 200 simulations (each black line) with a cutoff of at least 7 days from symptom onset to hospitalisation for defining a hospital-acquired case. The top two rows show the variation in community-onset, hospital-linked infections (first row) and subsequent cases (second row) at low, mean and high values of onward transmission (*R =* 0.76, 0.8, 0.84). The third row shows the counterfactual: the number of hospitalised cases there would be predicted to be without any hospital-acquisition of SARS-CoV-2, alongside the community-onset, community-acquired (“COCA”) and community-onset, hospital-acquired (“COHA) case estimates. The final row shows the same variation shown in Figure S8: the total number of unidentified infections and the “missed” subset of these (“missed” due to discharge prior to symptom onset). Note the variation in the y axis values.

We decided to use 200 simulations as above approximately 150 simulations the output for key parameters (shown in Figure S12) stabilised.

**
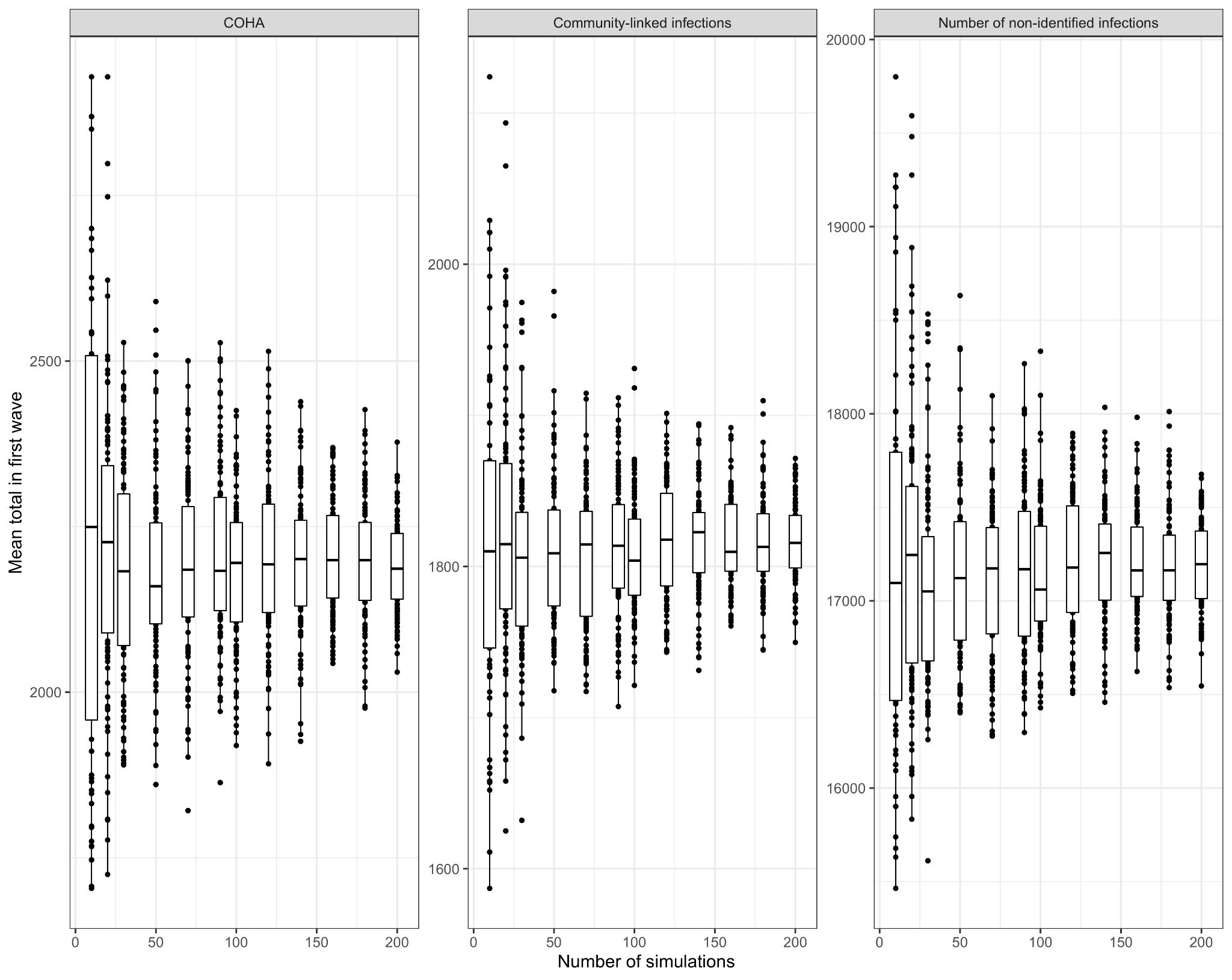
**

**Figure S12:** Boxplot of mean total value of key outcome variables over the “first wave” (to 31st July 2020) against the number of simulations. Left is “community onset, hospital-acquired” cases (COHA), middle are community-linked infections and right is the number of unidentified infections.

**Conclusion**

Uncertainty in our estimates was generated from sampling from a range of natural history distributions and the length of stay data. As we had data from SUS on the latter for a large number of non-COVID patients, we had little ambiguity in this key parameter for estimating the proportion of hospital-acquired infections identified. Moreover, much of the uncertainty was in the timing of events (symptom onset 2 or 5 days from infection for example), which, when aggregated over a 7-month period had little impact on the final aggregated results.

**References**

1. Knight GM. Supporting Github repository [Internet]. Github; 2021. Available from: https://github.com/gwenknight/hai_first_wave.git
