# Supplementary material for: The contribution of hospital-acquired infections to the COVID-19 epidemic in England in the first half of 2020
Source: BMC Infect Dis. 2022 Jun 18;22:556. doi: 10.1186/s12879-022-07490-4 (PMC9206097; doi:10.1186/s12879-022-07490-4)
Supplement: Supplementary file 11 — Additional file 11. Additional results. [file 12879_2022_7490_MOESM11_ESM.docx]

**Additional File 11: Additional results**

- **Figure 5 additional analysis**
- **Table S3**: Additional reported results
- **Table S4**: Estimated percentage of “community onset, community acquired” infections that would be re-classified as “community onset, hospital acquired” infections
- **Table S5**: Estimated number of community onset, hospital-linked cases
- **Figure S13:** Impact of 1 vs 5 day discharge before associated identified hospital case
- **Figure S14:** Impact of R value variation over time (not just aggregated)

**Figure 5 additional analysis**

- Of all hospital patients who had a SARS-CoV-2 infection some time during their stay, 29.6% (28.9%, 30.5%) were hospital-acquired (E/(A+D), Figure 5).
- With the addition of hospital-linked infections, out of all hospital patients with a SARS-CoV-2 infection, 31.5% (30.6%, 32.4%) were estimated to have acquired their infection in hospitals or were hospital-linked ((E+F)/(A+D), Figure 5).

| **Estimate** | **Cutoff** |  |  |  |
| --- | --- | --- | --- | --- |
|  | **7** | **14** | **4** | **Details** |
| “hospital-onset, hospital-acquired” identified cases across acute English Trusts up to the 31st July 2020 | 6,640 | 4,440 | 7,830 | From adjusted CO-CIN |
| unidentified hospital-acquired infections | 20,000 (19,200, 21,100) | 29,000 (28,400, 29,600) | 17,500 (16,000, 19,300) | mean; 95% range over 200 simulations |
| Percentage of “community-onset, community-acquired” that should be classified as “community-onset, hospital-acquired” | 2.1% (1.7%, 2.6%) | 2.6% (2.1%, 3.1%) | 2.1% (1.7%, 2.6%) | mean; 95% range over 200 simulations: |
| “community-onset, hospital-linked” cases | 1,600 (1,600, 1,700) | 2,100 (2,000, 2,200) | 1,600 (1,400, 1,700) | For the time varying *R* value mean; 95% range over 600 simulations |

**Table S3:** Estimated additional main results for 14 and 4 day cut-offs in line with 7 day values in main text

**Table S4:** Estimated mean and 95% quantile range over 200 simulations of the percentage of “community onset, community acquired” infections that would be re-classified as “community onset, hospital acquired” infections under different *R* values, hospital-acquired (HA) definition cutoffs (if symptom onset starts this many days from admission), discharge times from associated hospital-acquired case for unidentified hospital-acquired infection and scenarios for symptom onset to hospitalisation.

| **R value (0.8, 1.2, *rt*)** | **HA definition cutoff (5,8,15)** | **Discharge time for unidentified hospital-acquired infection**  **(1 or 5)** | **Symptom onset to hospitalisation scenario**  **(1-3)** | **Mean** | **95% quantile range** | |
| --- | --- | --- | --- | --- | --- | --- |
| 0.8 | 5 | 5 | 1 | 2.1 | 1.6 | 2.5 |
| 0.8 | 5 | 5 | 2 | 2.1 | 1.6 | 2.5 |
| 0.8 | 5 | 5 | 3 | 2.1 | 1.6 | 2.5 |
| 0.8 | 8 | 5 | 1 | 2.1 | 1.7 | 2.6 |
| 0.8 | 8 | 5 | 2 | 2.1 | 1.7 | 2.6 |
| 0.8 | 8 | 5 | 3 | 2.1 | 1.7 | 2.6 |
| 0.8 | 15 | 5 | 1 | 2.6 | 2.1 | 3.1 |
| 0.8 | 15 | 5 | 2 | 2.6 | 2.1 | 3.1 |
| 0.8 | 15 | 5 | 3 | 2.6 | 2.1 | 3.1 |
| 1.2 | 5 | 5 | 1 | 2.1 | 1.6 | 2.5 |
| 1.2 | 5 | 5 | 2 | 2.1 | 1.6 | 2.5 |
| 1.2 | 5 | 5 | 3 | 2.1 | 1.6 | 2.5 |
| 1.2 | 8 | 5 | 1 | 2.1 | 1.7 | 2.6 |
| 1.2 | 8 | 5 | 2 | 2.1 | 1.7 | 2.6 |
| 1.2 | 8 | 5 | 3 | 2.1 | 1.7 | 2.6 |
| 1.2 | 15 | 5 | 1 | 2.6 | 2.1 | 3.1 |
| 1.2 | 15 | 5 | 2 | 2.6 | 2.1 | 3.1 |
| 1.2 | 15 | 5 | 3 | 2.6 | 2.1 | 3.1 |
| rt | 5 | 5 | 1 | 2.1 | 1.6 | 2.6 |
| rt | 5 | 5 | 2 | 2.1 | 1.6 | 2.5 |
| rt | 5 | 5 | 3 | 2.1 | 1.6 | 2.6 |
| rt | 8 | 5 | 1 | 2.1 | 1.7 | 2.6 |
| rt | 8 | 5 | 2 | 2.1 | 1.7 | 2.6 |
| rt | 8 | 5 | 3 | 2.1 | 1.7 | 2.6 |
| rt | 15 | 5 | 1 | 2.6 | 2.1 | 3.1 |
| rt | 15 | 5 | 2 | 2.6 | 2.1 | 3.1 |
| rt | 15 | 5 | 3 | 2.6 | 2.1 | 3.1 |
| 0.8 | 5 | 1 | 1 | 2.1 | 1.6 | 2.5 |
| 0.8 | 5 | 1 | 2 | 2.1 | 1.6 | 2.5 |
| 0.8 | 5 | 1 | 3 | 2.1 | 1.6 | 2.5 |
| 0.8 | 8 | 1 | 1 | 2.1 | 1.7 | 2.6 |
| 0.8 | 8 | 1 | 2 | 2.1 | 1.7 | 2.6 |
| 0.8 | 8 | 1 | 3 | 2.1 | 1.7 | 2.6 |
| 0.8 | 15 | 1 | 1 | 2.6 | 2.1 | 3.1 |
| 0.8 | 15 | 1 | 2 | 2.6 | 2.1 | 3.1 |
| 0.8 | 15 | 1 | 3 | 2.6 | 2.1 | 3.1 |
| 1.2 | 5 | 1 | 1 | 2.1 | 1.6 | 2.6 |
| 1.2 | 5 | 1 | 2 | 2.1 | 1.6 | 2.5 |
| 1.2 | 5 | 1 | 3 | 2.1 | 1.6 | 2.5 |
| 1.2 | 8 | 1 | 1 | 2.1 | 1.7 | 2.6 |
| 1.2 | 8 | 1 | 2 | 2.1 | 1.7 | 2.6 |
| 1.2 | 8 | 1 | 3 | 2.1 | 1.7 | 2.6 |
| 1.2 | 15 | 1 | 1 | 2.6 | 2.1 | 3.1 |
| 1.2 | 15 | 1 | 2 | 2.6 | 2.1 | 3.1 |
| 1.2 | 15 | 1 | 3 | 2.6 | 2.1 | 3.1 |
| rt | 5 | 1 | 1 | 2.1 | 1.6 | 2.5 |
| rt | 5 | 1 | 2 | 2.1 | 1.6 | 2.5 |
| rt | 5 | 1 | 3 | 2.1 | 1.6 | 2.5 |
| rt | 8 | 1 | 1 | 2.1 | 1.7 | 2.6 |
| rt | 8 | 1 | 2 | 2.1 | 1.7 | 2.6 |
| rt | 8 | 1 | 3 | 2.1 | 1.7 | 2.6 |
| rt | 15 | 1 | 1 | 2.6 | 2.1 | 3.1 |
| rt | 15 | 1 | 2 | 2.6 | 2.1 | 3.1 |
| rt | 15 | 1 | 3 | 2.6 | 2.1 | 3.1 |

**Table S5:** Estimated mean and 95% quantile range over 200 simulations number and percentage contribution of “community onset, hospital linked cases” under different *R* values, hospital-acquired (HA) definition cutoffs (if symptom onset starts this many days from admission), discharge times from associated hospital-acquired case for unidentified hospital-acquired infection and scenarios for symptom onset to hospitalisation.

|  |  |  |  | **Number of infections** | | | **Proportion of community onset community acquired cases** | | |
| --- | --- | --- | --- | --- | --- | --- | --- | --- | --- |
| **R value**  **(0.8, 1.2, *rt*)** | **HA definition cutoff**  **(5,8,15)** | **Discharge time for unidentified hospital-acquired infection**  **(1 or 5)** | **Symptom onset to hospitalisation scenario**  **(1-3)** | **Mean** | **95% quantile range** | | **Mean** | **95% quantile range** | |
| 0.8 | 5 | 5 | 1 | 1000 | 800 | 1100 | 1.3 | 1.2 | 1.5 |
| 0.8 | 5 | 5 | 2 | 1000 | 800 | 1100 | 1.3 | 1.2 | 1.6 |
| 0.8 | 5 | 5 | 3 | 1000 | 800 | 1100 | 1.3 | 1.2 | 1.5 |
| 0.8 | 8 | 5 | 1 | 1000 | 900 | 1100 | 1.4 | 1.2 | 1.5 |
| 0.8 | 8 | 5 | 2 | 1000 | 900 | 1100 | 1.4 | 1.3 | 1.5 |
| 0.8 | 8 | 5 | 3 | 1000 | 900 | 1100 | 1.4 | 1.2 | 1.5 |
| 0.8 | 15 | 5 | 1 | 1300 | 1200 | 1400 | 1.7 | 1.6 | 1.8 |
| 0.8 | 15 | 5 | 2 | 1300 | 1200 | 1400 | 1.7 | 1.6 | 1.8 |
| 0.8 | 15 | 5 | 3 | 1300 | 1200 | 1400 | 1.7 | 1.6 | 1.8 |
| 1.2 | 5 | 5 | 1 | 2600 | 2300 | 2900 | 3.6 | 3.2 | 4.1 |
| 1.2 | 5 | 5 | 2 | 2600 | 2300 | 2900 | 3.6 | 3.3 | 4.1 |
| 1.2 | 5 | 5 | 3 | 2600 | 2300 | 3000 | 3.7 | 3.3 | 4.2 |
| 1.2 | 8 | 5 | 1 | 2700 | 2500 | 3000 | 3.8 | 3.5 | 4.1 |
| 1.2 | 8 | 5 | 2 | 2700 | 2500 | 3000 | 3.8 | 3.5 | 4.1 |
| 1.2 | 8 | 5 | 3 | 2700 | 2500 | 3000 | 3.8 | 3.5 | 4.1 |
| 1.2 | 15 | 5 | 1 | 3400 | 3200 | 3700 | 4.6 | 4.3 | 4.9 |
| 1.2 | 15 | 5 | 2 | 3400 | 3300 | 3700 | 4.6 | 4.4 | 4.9 |
| 1.2 | 15 | 5 | 3 | 3500 | 3200 | 3700 | 4.6 | 4.3 | 5 |
| rt | 5 | 5 | 1 | 1600 | 1400 | 1700 | 2.2 | 2 | 2.4 |
| rt | 5 | 5 | 2 | 1600 | 1400 | 1700 | 2.2 | 2 | 2.4 |
| rt | 5 | 5 | 3 | 1600 | 1400 | 1700 | 2.2 | 2 | 2.4 |
| rt | 8 | 5 | 1 | 1600 | 1600 | 1700 | 2.3 | 2.1 | 2.4 |
| rt | 8 | 5 | 2 | 1600 | 1500 | 1700 | 2.3 | 2.1 | 2.4 |
| rt | 8 | 5 | 3 | 1600 | 1500 | 1800 | 2.3 | 2.1 | 2.4 |
| rt | 15 | 5 | 1 | 2100 | 2000 | 2200 | 2.8 | 2.7 | 2.9 |
| rt | 15 | 5 | 2 | 2100 | 2000 | 2200 | 2.8 | 2.7 | 2.9 |
| rt | 15 | 5 | 3 | 2100 | 2000 | 2200 | 2.8 | 2.7 | 2.9 |
| 0.8 | 5 | 1 | 1 | 900 | 800 | 1100 | 1.3 | 1.2 | 1.5 |
| 0.8 | 5 | 1 | 2 | 1000 | 800 | 1100 | 1.3 | 1.2 | 1.5 |
| 0.8 | 5 | 1 | 3 | 1000 | 800 | 1100 | 1.3 | 1.2 | 1.5 |
| 0.8 | 8 | 1 | 1 | 1000 | 900 | 1100 | 1.4 | 1.3 | 1.5 |
| 0.8 | 8 | 1 | 2 | 1000 | 900 | 1100 | 1.4 | 1.3 | 1.5 |
| 0.8 | 8 | 1 | 3 | 1000 | 900 | 1100 | 1.4 | 1.3 | 1.5 |
| 0.8 | 15 | 1 | 1 | 1300 | 1200 | 1400 | 1.7 | 1.6 | 1.8 |
| 0.8 | 15 | 1 | 2 | 1300 | 1200 | 1400 | 1.7 | 1.6 | 1.8 |
| 0.8 | 15 | 1 | 3 | 1300 | 1200 | 1400 | 1.7 | 1.6 | 1.8 |
| 1.2 | 5 | 1 | 1 | 2600 | 2300 | 2900 | 3.6 | 3.2 | 4.1 |
| 1.2 | 5 | 1 | 2 | 2600 | 2300 | 2900 | 3.6 | 3.2 | 4.1 |
| 1.2 | 5 | 1 | 3 | 2600 | 2300 | 2900 | 3.6 | 3.3 | 4.1 |
| 1.2 | 8 | 1 | 1 | 2700 | 2500 | 2900 | 3.8 | 3.5 | 4.1 |
| 1.2 | 8 | 1 | 2 | 2700 | 2500 | 3000 | 3.8 | 3.5 | 4.1 |
| 1.2 | 8 | 1 | 3 | 2700 | 2500 | 3000 | 3.8 | 3.5 | 4.1 |
| 1.2 | 15 | 1 | 1 | 3400 | 3200 | 3700 | 4.6 | 4.3 | 4.9 |
| 1.2 | 15 | 1 | 2 | 3400 | 3200 | 3700 | 4.6 | 4.3 | 4.9 |
| 1.2 | 15 | 1 | 3 | 3400 | 3200 | 3700 | 4.6 | 4.3 | 4.9 |
| *rt* | 5 | 1 | 1 | 1300 | 1200 | 1500 | 1.9 | 1.7 | 2.1 |
| *rt* | 5 | 1 | 2 | 1300 | 1200 | 1500 | 1.9 | 1.7 | 2.1 |
| *rt* | 5 | 1 | 3 | 1300 | 1200 | 1500 | 1.9 | 1.7 | 2.1 |
| *rt* | 8 | 1 | 1 | 1400 | 1300 | 1500 | 1.9 | 1.8 | 2.1 |
| *rt* | 8 | 1 | 2 | 1400 | 1300 | 1500 | 1.9 | 1.8 | 2.1 |
| *rt* | 8 | 1 | 3 | 1400 | 1300 | 1500 | 1.9 | 1.8 | 2.1 |
| *rt* | 15 | 1 | 1 | 1800 | 1700 | 1900 | 2.4 | 2.2 | 2.5 |
| *rt* | 15 | 1 | 2 | 1800 | 1700 | 1900 | 2.4 | 2.2 | 2.5 |
| *rt* | 15 | 1 | 3 | 1800 | 1700 | 1900 | 2.4 | 2.2 | 2.5 |

**Impact of 1 - 5 day discharge**

**
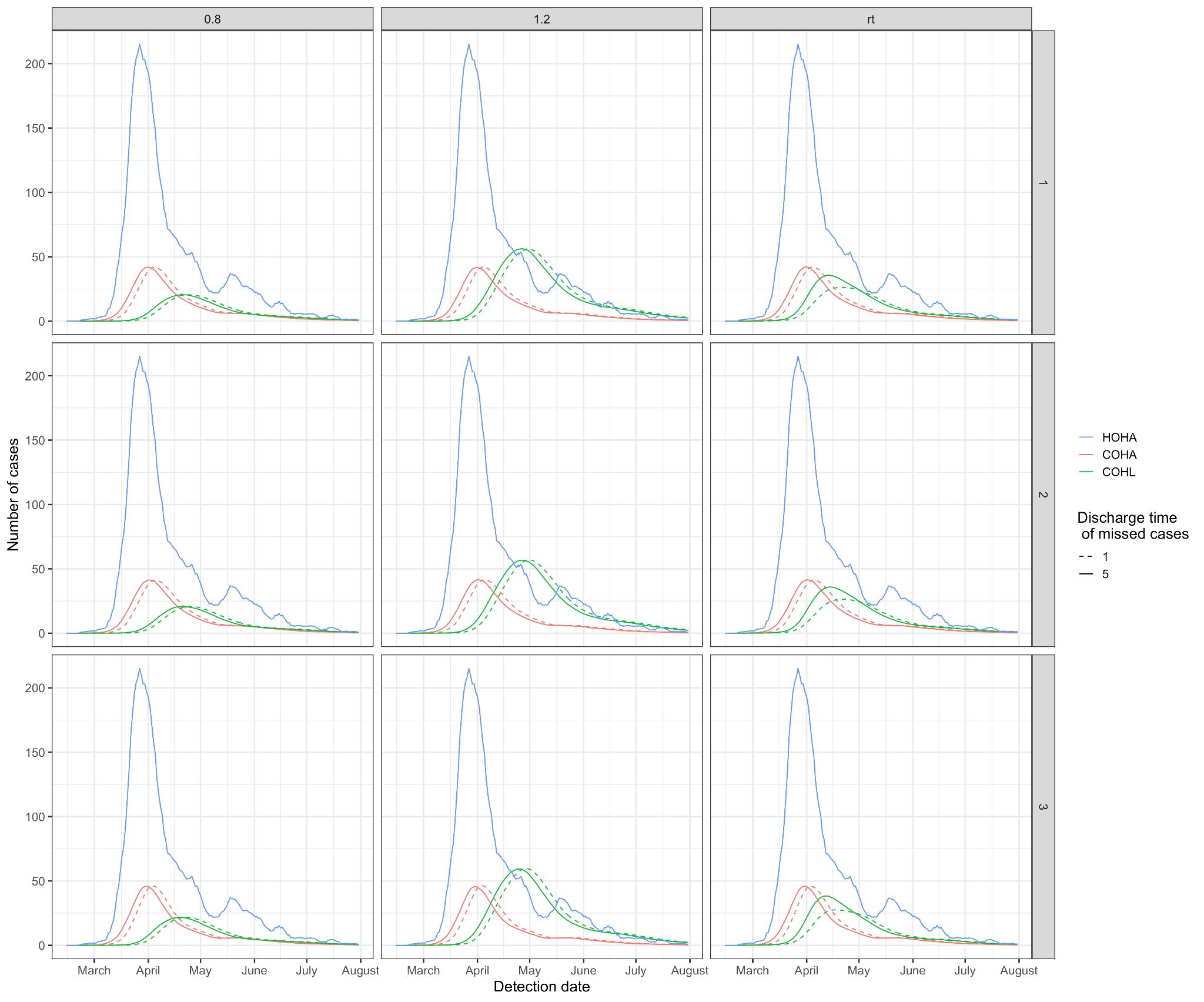
**

**Figure S13: The impact of discharging missed cases 5 days (solid line, baseline) or 1 day (dashed line) before the associated identified hospital-acquired case at a cut-off threshold of 7 days from admission across different *R* values (columns) and Scenarios (rows) of symptom onset to hospitalisation. This is for “hospital-onset, hospital-acquired” (HOHA, blue), “community-onset, hospital-acquired” (COHA, red) and “community-onset, hospital-linked” (COHL, green) cases**

As shown in Figure S13, there is a minimal impact of varying the day of discharge of missed cases, except for the “community-onset, hospital-linked” (COHL) cases when using the time varying *R* estimates (“rt”). Cumulatively, up to the end of July 2020, this results in a less than 0.001% change in the number of “community-onset, community-acquired” cases but a ~30% higher number of “community-onset, hospital-linked” cases when using the time varying *R* estimates (“rt”) and a 5 day discharge. This is due to a synergistic impact of the missed infections entering the community at peak R value (before early April).

**Impact of R value variation**

**
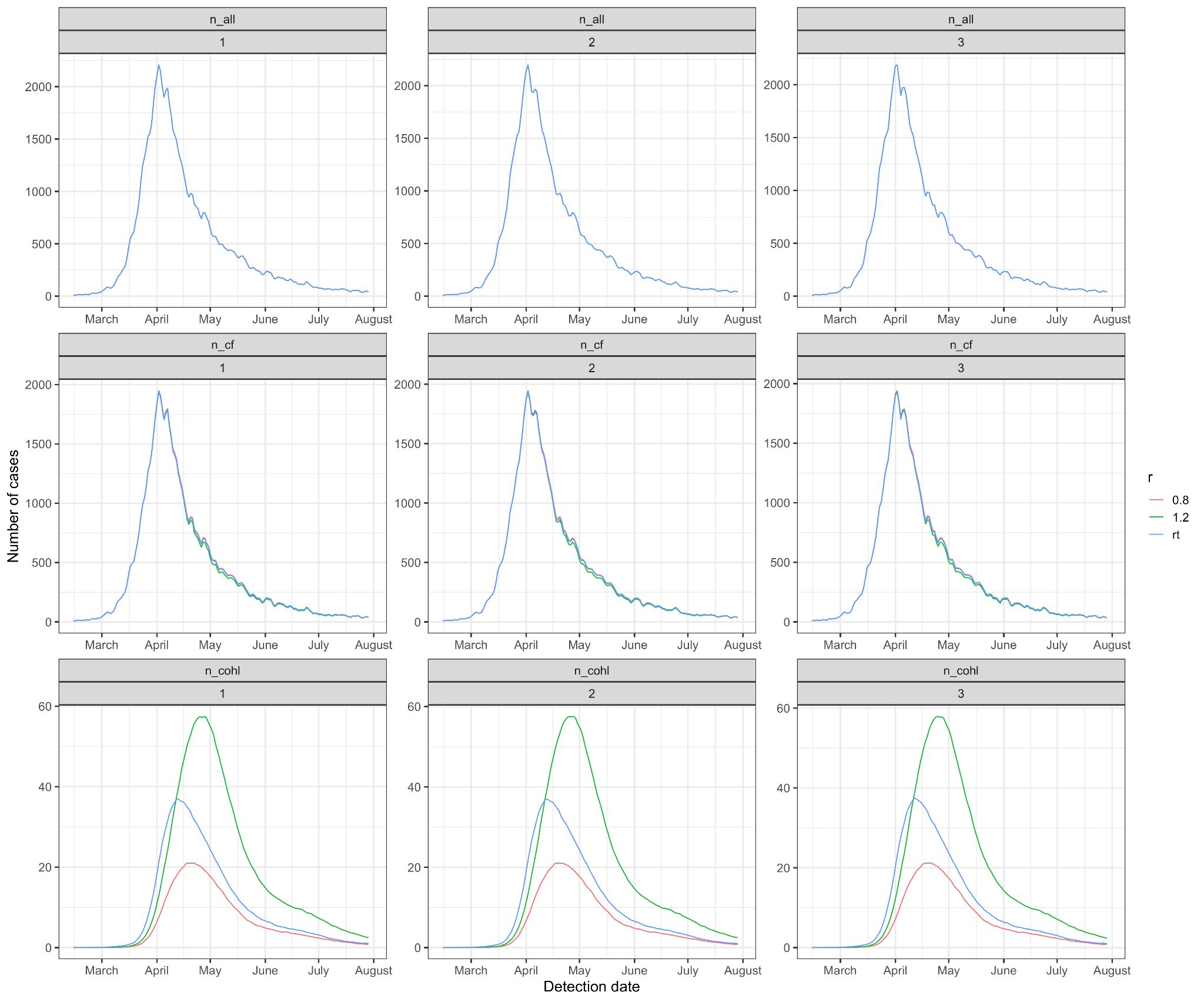
**

**Figure S14: Time series of all hospitalised, counter-factual and community onset, hospital-linked cases under different onward transmission (*R*) values (median values shown here, colours). This is for a cutoff of 7 days from admission for the hospital-acquired definition and for the three scenarios (columns) for symptom onset to hospitalisation.**
