# Supplementary material for: The contribution of hospital-acquired infections to the COVID-19 epidemic in England in the first half of 2020
Source: BMC Infect Dis. 2022 Jun 18;22:556. doi: 10.1186/s12879-022-07490-4 (PMC9206097; doi:10.1186/s12879-022-07490-4)
Supplement: Supplementary file 12 — Additional file 12. Grouped trust level analysis. [file 12879_2022_7490_MOESM12_ESM.docx]

**Additional File 12: Grouped Trust level analysis**

**Method:** We applied the same analysis (shown in Figure 2) at the individual acute Trust level (n = 126) and then aggregated the results. We performed this analysis for all three cutoffs, three *R* values and for the first symptom onset to hospitalisation scenario with 50 simulations for each of the 126 Trusts to generate uncertainty ranges.

**Results:**

4 Trusts had no nosocomial cases recorded in the data over this time period. Two Trusts had no nosocomial cases recorded when using a 14 day cutoff for definition of a nosocomial case.

The proportion missed each week varied over trusts with a mean of 29% and a range between 0 and 88% over 50 simulations and all weeks and Trusts. 0.2% of the proportion detected estimates were zero.

Comparing the aggregated England setting (data pooled before doing analysis) to the grouped individual Trust (analysis performed at the Trust level and then aggregated) analysis shows similar results but the levels from the individual Trust analysis is higher (Table S5). Some variation would be expected due to rounding e.g. of the number of missed infections from the identified number of hospital-acquired cases. At the baseline cutoff of symptom onset 8 or more days from admission, the variation is relatively small, but it increases at a 15 or more days from admission cutoff, especially for “community-onset, hospital-linked” cases. The similarity in key indicators is shown in Figure S15-17 below. Using the grouped individual Trust analysis predicts that 25.5% (24.6%, 26.4%) of identified COVID-19 cases in hospitals were hospital-acquired, higher than the level predicted from the aggregated England setting: 20.1% (19.2%, 20.7%).

**Comparison and interpretation:** The proportion identified is predicted to be very small when there are few hospital-onset, hospital-acquired (HOHA) cases, as is often the case when doing the analysis at the individual trust level. Using the Bayesian framework to infer the total number of hospital-acquired infections (“trials”) results in higher numbers (~50%) for the estimated number of unidentified hospital-acquired infections and hence onward case estimates (COHL / COHA). For example, 1 HOHA case, with a proportion detected of 0.005, is predicted to be linked to 524 hospital-acquired infections. However, 7 HOHA cases, with the same proportion detected, results in a predicted 1450 infections: an increase of 3x infections instead of 7x as might be expected from the increase in HOHA. We believe that the analysis at the Trust level suffers from issues of small numbers and issues with using the empiric length of stay distributions. This leads to unrealistically small proportions detected and hence inflation to a greater number of missed infections.

***Table S5: Comparison of England level vs. grouped trusts analysis for varying cutoff and R values for three key indicators under the first scenario for symptom onset to hospital admission. The values presented are the mean and 95% quantile over 200 simulations for the aggregated England setting and over 50 simulations for each Trust. Bold values are those with the baseline cutoff.***

| **Setting** | **Cutoff for defining hospital-acquired (symptom onset on this day or later after admission)** | ***R* value** | **Number of hospital-onset hospital-acquired identified cases (HOHA)** | **Number of unidentified hospital-acquired infections** | **Number of community-onset hospital-linked cases (COHL)** |
| --- | --- | --- | --- | --- | --- |
|  |  |  |  |  |  |
| ENG | 5 | 0.8 | 7,800 (7,800, 7,800) | 17,400 (16,100, 19,100) | 1,000 (900, 1,100) |
| grouped |  | 0.8 | 7,400 (7,400, 7,400) | 24,700 (22,500, 27,100) | 1,500 (1,400, 1,700) |
| ENG |  | 1.2 | 7,800 (7,800, 7,800) | 17,400 (16,100, 19,100) | 2,600 (2,400, 2,900) |
| grouped |  | 1.2 | 7,400 (7,400, 7,400) | 24,700 (22,500, 27,100) | 3,400 (3,100, 3,900) |
| ENG |  | rt | 7,800 (7,800, 7,800) | 17,400 (16,100, 19,100) | 1,600 (1,500, 1,700) |
| grouped |  | rt | 7,400 (7,400, 7,400) | 24,700 (22,500, 27,100) | 2,400 (2,200, 2,600) |
| **ENG** | **8** | **0.8** | **6,600 (6,600, 6,600)** | **20,000 (19,300, 21,000)** | **1,000 (900, 1,100)** |
| **grouped** |  | **0.8** | **6,200 (6,200, 6,200)** | **29,200 (28,100, 30,800)** | **1,700 (1,500, 1,800)** |
| **ENG** |  | **1.2** | **6,600 (6,600, 6,600)** | **20,000 (19,300, 21,000)** | **2,800 (2,500, 3,100)** |
| **grouped** |  | **1.2** | **6,200 (6,200, 6,200)** | **29,200 (28,100, 30,800)** | **3,800 (3,500, 4,200)** |
| **ENG** |  | **rt** | **6,600 (6,600, 6,600)** | **20,000 (19,300, 21,000)** | **1,700 (1,600, 1,800)** |
| **grouped** |  | **rt** | **6,200 (6,200, 6,200)** | **29,200 (28,100, 30,800)** | **2,500 (2,400, 2,700)** |
| ENG | 15 | 0.8 | 4,400 (4,400, 4,400) | 29,100 (28,500, 29,700) | 1,300 (1,200, 1,400) |
| grouped |  | 0.8 | 4,000 (4,000, 4,000) | 55,700 (51,500, 62,900) | 2,600 (2,400, 3,000) |
| ENG |  | 1.2 | 4,400 (4,400, 4,400) | 29,100 (28,500, 29,700) | 3,500 (3,300, 3,800) |
| grouped |  | 1.2 | 4,000 (4,000, 4,000) | 55,700 (51,500, 62,900) | 6,600 (5,900, 7,400) |
| ENG |  | rt | 4,400 (4,400, 4,400) | 29,100 (28,500, 29,700) | 2,100 (2,000, 2,200) |
| grouped |  | rt | 4,000 (4,000, 4,000) | 55,700 (51,500, 62,900) | 4,100 (3,700, 4,500) |

**
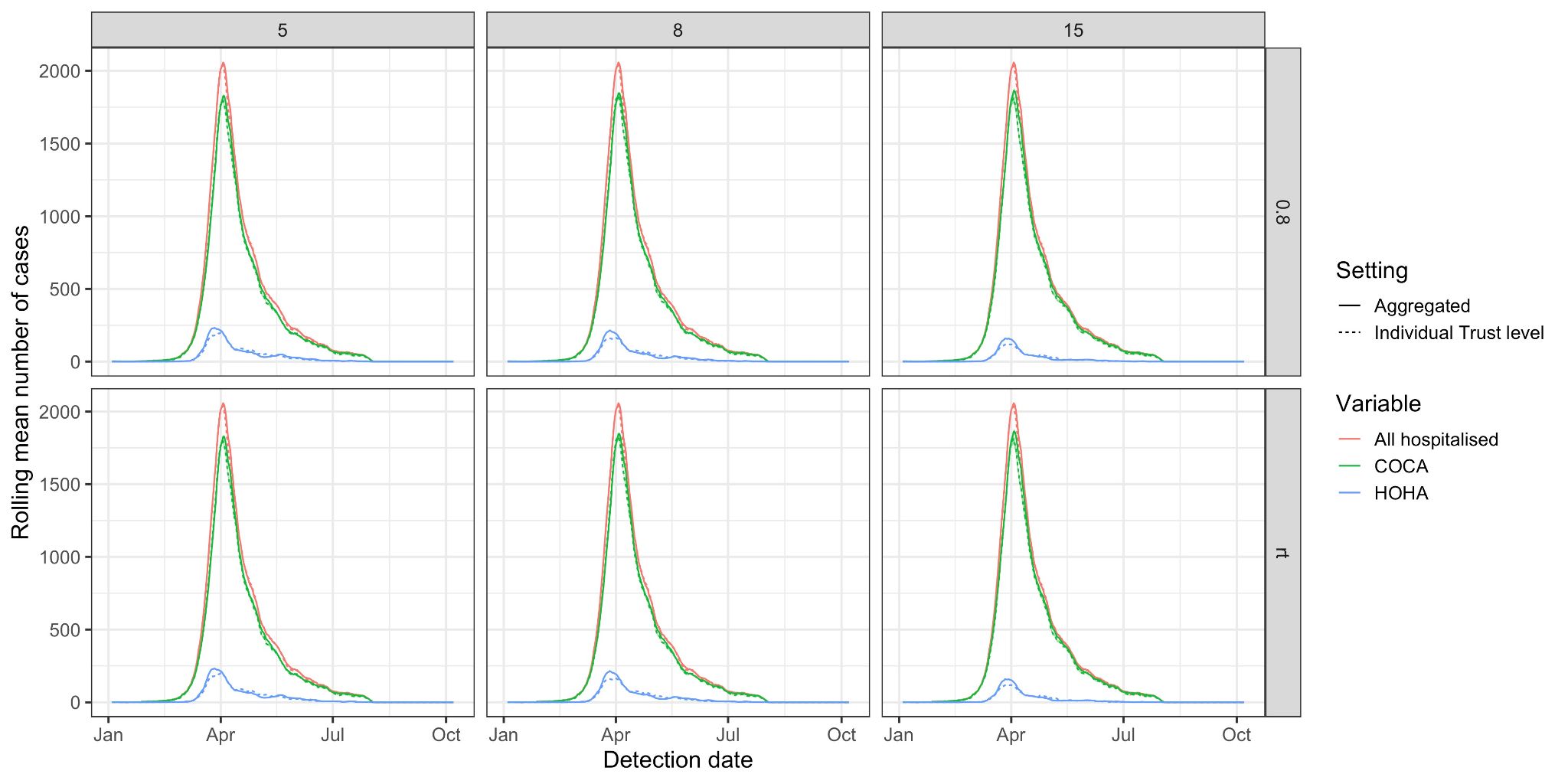
**

**Figure S15: For the first symptom onset to hospitalisation scenario, there is little variation in the output of key case numbers (all hospitalised (red), community-onset, community-acquired (COCA, green) and hospital-onset, hospital-acquired (HOHA, blue)) if the analysis is performed on the aggregated England setting level (solid line, baseline) or at the individual Trust level and then aggregated (dashed line). The line here is the mean over 200 simulations for the aggregated England setting (50 simulations per Trust for the individual Trust analysis) and 95% range in shaded area.**

**
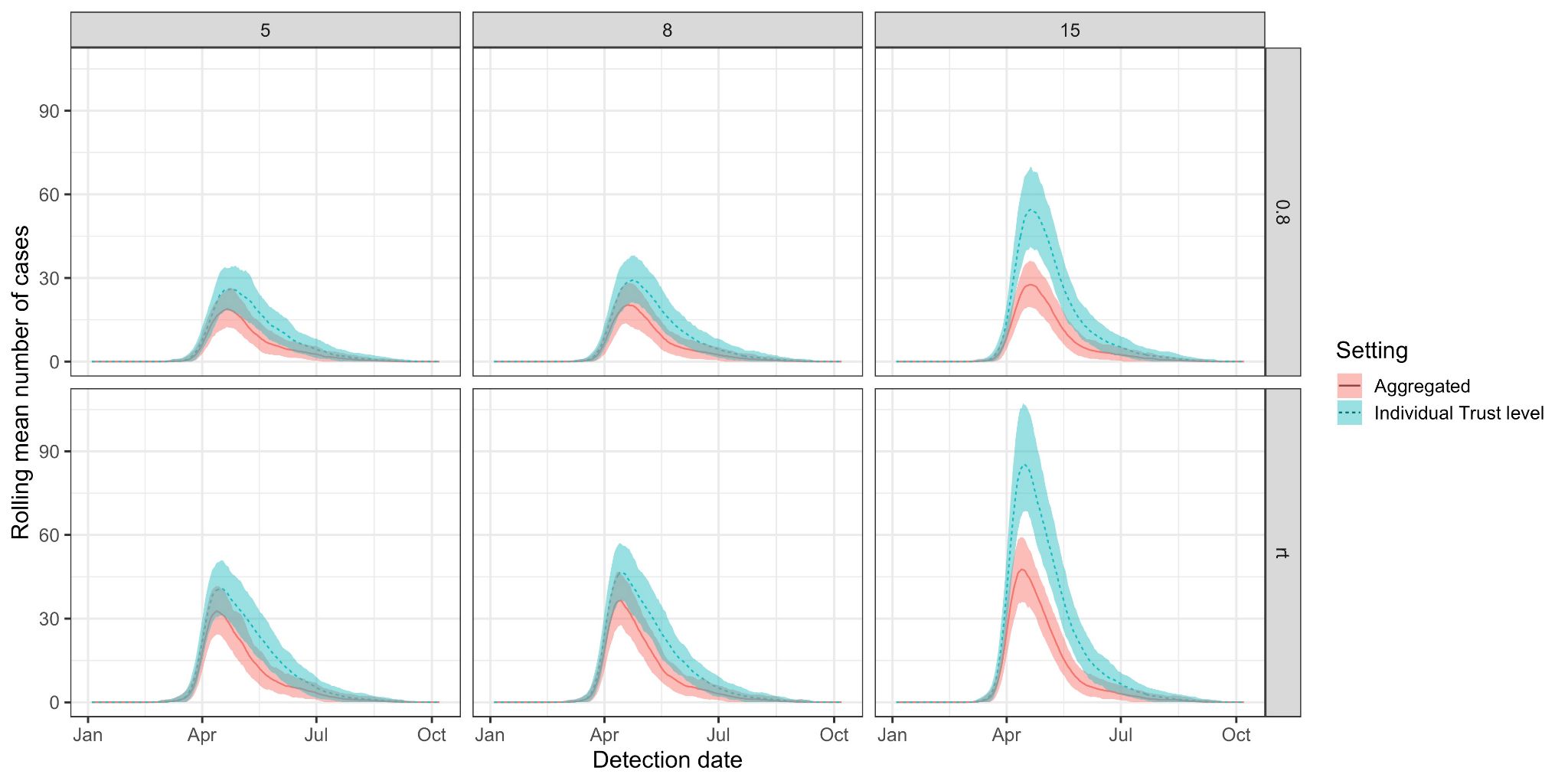
**

**Figure S16: For the first symptom onset to hospitalisation scenario, there is some variation in the number of community-onset, hospital-linked cases if the analysis is performed on the aggregated England setting level (solid line, baseline, red) or at the individual Trust level and then aggregated (dashed line, blue). The line here is the mean over 200 simulations for the aggregated England setting (50 simulations per Trust for the individual Trust analysis) and 95% range given in the shaded area.**

**
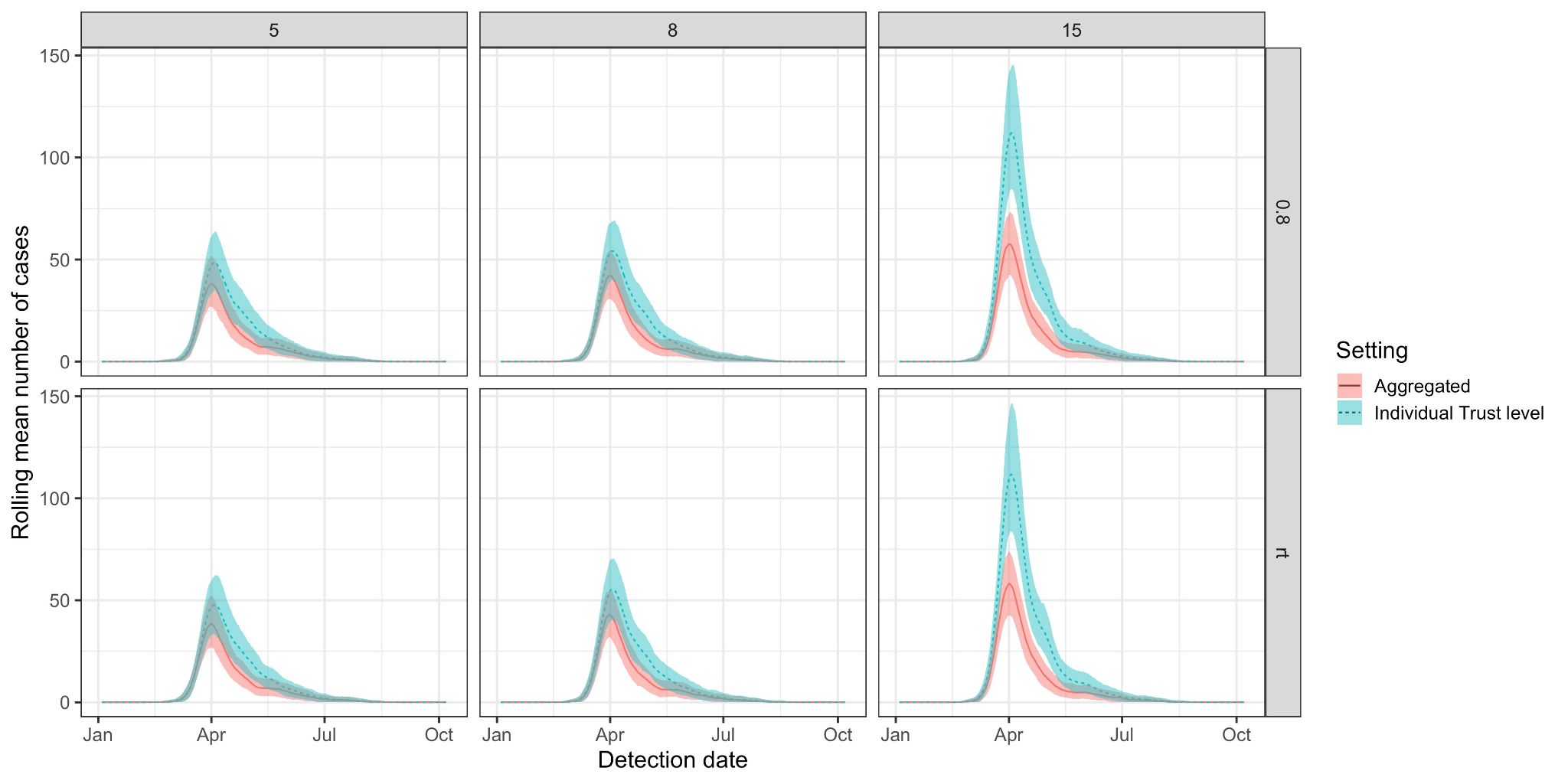
**

**Figure S17: For the first symptom onset to hospitalisation scenario, there is some variation in the number of community-onset, hospital-acquired cases if the analysis is performed on the aggregated England setting level (solid line, baseline, red) or at the individual Trust level and then aggregated (dashed line, blue). The line here is the mean over 200 simulations for the aggregated England setting (50 simulations per Trust for the individual Trust analysis) and 95% range given in the shaded area**
